# Effects of Detergent on α-Synuclein Structure: A Native MS-Ion Mobility Study

**DOI:** 10.3390/ijms21217884

**Published:** 2020-10-23

**Authors:** Rani Moons, Renate van der Wekken-de Bruijne, Stuart Maudsley, Filip Lemière, Anne-Marie Lambeir, Frank Sobott

**Affiliations:** 1 Biomolecular and Analytical Mass Spectrometry Group, University of Antwerp, 2020 Antwerp, Belgium; rani.moons@uantwerpen.be (R.M.); renatedebruijne@hotmail.com (R.v.d.W.-d.B.); filip.lemiere@uantwerpen.be (F.L.); 2 Receptor Biology Lab, University of Antwerp, 2610 Antwerp, Belgium; Stuart.maudsley@uantwerpen.be; 3 Laboratory of Medical Biochemistry, University of Antwerp, 2610 Antwerp, Belgium; anne-marie.lambeir@uantwerpen.be; 4 Astbury Centre for Structural Molecular Biology, University of Leeds, Leeds LS2 9JT, UK; 5 School of Molecular and Cellular Biology, University of Leeds, Leeds LS2 9JT, UK

**Keywords:** ion mobility, mass spectrometry, α-synuclein, intrinsically disordered protein, detergent micelles, membrane interaction, protein conformation, ligand binding, electrospray ionization

## Abstract

The intrinsically disordered protein α-synuclein plays a major role in Parkinson’s disease. The protein can oligomerize resulting in the formation of various aggregated species in neuronal cells, leading to neurodegeneration. The interaction of α-synuclein with biological cell membranes plays an important role for specific functions of α-synuclein monomers, e.g., in neurotransmitter release. Using different types of detergents to mimic lipid molecules present in biological membranes, including the presence of Ca^2+^ ions as an important structural factor, we aimed to gain an understanding of how α-synuclein interacts with membrane models and how this affects the protein conformation and potential oligomerization. We investigated detergent binding stoichiometry, affinity and conformational changes of α-synuclein taking detergent concentration, different detergent structures and charges into account. With native nano-electrospray ionization ion mobility-mass spectrometry, we were able to detect unique conformational patterns resulting from binding of specific detergents to α-synuclein. Our data demonstrate that α-synuclein monomers can interact with detergent molecules irrespective of their charge, that protein-micelle interactions occur and that micelle properties are an important factor.

## 1. Introduction

The protein alpha synuclein (α-syn) is abundant in the human body, particularly in neurons where it accounts for around 1% of the full neuronal proteome [1]. α-syn is a 140 amino acid (aa), intrinsically disordered protein that lacks a well-defined globular structure, can self-aggregate and plays an important pathogenic role in Parkinson’s disease (PD) [2,3,4].

Three distinct regions can be distinguished within the primary amino acid sequence. The N-terminal region (aa 1–60) contains a high number of positively charged residues. In solution this region adopts diverse extended and more compact states, but can form an amphipathic alpha helix when interacting and binding to membranes or membrane mimicking environments [5,6]. Familial mutations in α-syn known to cause PD are found in this part of the protein [7,8,9,10]. The middle region, aa 61–95, is known as the Non-Amyloid-β Component (NAC) domain. This region contains a high number of hydrophobic residues and can form β-sheet amyloid structures through interaction with NAC regions in other α-syn molecules. The third region, the C-terminal part, contains many negatively charged amino acids and retains a disordered structure. Most post-translational modifications (PTMs) as well as binding sites for various cations and truncation sites are located in this part of the protein [5,11,12]. With a pI of 4.67 the overall protein is negatively charged at physiological pH.

α-syn has been described to form intermediate, oligomeric structures such as protofibrils or gel-like condensates [13]. These intermediate structures can aggregate further into mature fibrils with a characteristic β-sheet structure, which are found in Lewy body inclusions in neuronal cells of PD patients [14,15].

The interactions between specific forms of α-syn, e.g., oligomers, and biological membranes recently gained considerable interest since aggregates of α-syn are thought to cause neuronal cell death, possibly by interacting with the cell membrane leading to a disturbance of the lipid arrangement or by pore formation. This proposed pathological α-syn mechanism presents a current and intensely-studied issue in neurodegenerative molecular biology [13,16,17]. It has also been shown that α-syn monomers can interact with vesicles in the intracellular environment, and that this interaction depends on the charge of the lipids and the curvature of the membrane [18,19]. Ca^2+^ is an important binding partner in the cellular environment that also plays a role in the interaction of α-syn with biological membranes. This metal ion, crucial for vesicular release-based neurotransmission regulation has also been shown to cause a conformational transition in α-syn and in this way trigger specific functions of the protein in vesicle trafficking pathways [20,21,22].

In vitro membrane-mimicking environments such as detergent micelles, amphipols, bicelles, nanodiscs, styrene maleic acid lipid particles (SMALPs) and small unilamellar vesicles (SUVs) are commonly used to study the structure of membrane proteins and their interaction with biological membranes [23,24,25,26]. While it is clear that there is an important interaction between α-syn and the presynaptic membrane, there is little detailed understanding at the molecular level under either physiological or pathological conditions. Is the charge of the membrane surface, or of individual lipids, the most important parameter? How is the conformational behaviour of α-syn monomers and oligomers altered and is this due to hydrophobic, or more specific electrostatic contacts with the membrane? We hypothesize that conformational changes at the monomer level may affect or even determine the further α-syn aggregation pathway, with biological membranes potentially playing an important role in this process, leading to different aggregate structures and corresponding physiological outcomes such as neurotoxicity [27].

Here we take a biophysical, in vitro approach, investigating how α-syn monomers interact with individual lipid-mimicking detergent molecules and what effects on α-syn structure these interactions induce. Detergents are amphiphilic organic molecules that possess a polar hydrophilic headgroup and a, usually longer, non-polar hydrophobic tail. Using a range of detergents we aim to represent the spectrum of lipids present in mammalian cells. The structure of detergents used in this study is analogous to lipids present in cell membranes, e.g., phosphatidylcholine, phosphatidylethanolamine and cholesterol [28,29,30]. Detergents which are dissolved in water above their critical micelle concentration (CMC) self-assemble into micelles, i.e., non-covalent clusters, the formation of which is driven by the hydrophobic effect, where hydrophobic parts of the detergent cluster together on the inside and hydrophilic groups are exposed to the aqueous solution outside [31]. In theory micelles are near spherical around the protein but the micelle can also form around specific hydrophobic patches of a protein, resulting in more asymmetric micelles with less organized rough outer surfaces [32]. Bile acid derivatives are a specific type of detergents which have structures that are more bean-shaped with a hydrophilic and a hydrophobic side, resulting in the formation of elliptic micelles [33].

Here we employ detergents as a model system to investigate the interactions, and possible resulting conformational changes and oligomerization, of α-syn monomers. We investigate if specific conformational effects can be attributed to a particular type of detergent, distinguishing between electrostatic effects or effects related to their hydrophobic nature. Detergent concentrations used represent free molecules (below CMC) and micelles (above CMC). Besides investigating the importance of the charge of the head group, we also examine effects of different hydrophobic chain lengths of the detergents.

Native nano-electrospray ionization coupled to ion mobility-mass spectrometry (nESI-IM-MS) enables a capacity to detect the interaction of detergents with the protein, the stoichiometry of binding and how this may be affected by the detergent concentration in relation to the CMC. With ion mobility, changes in the conformational behavior of α-syn monomers in the presence of, or binding to, a specific detergent can be detected as a heterogeneous conformational ensemble, rather than just showing averaged behavior. Conformations are discerned using their collision cross section (CCS) in Å², which is the rotationally averaged projection surface area and represents a global compactness parameter. Gradually activating the protein ions inside the mass spectrometer by increasing their internal energy, an approach known as Collision-Induced Unfolding (CIU), highlights different pathways and thresholds for structural transitions and gives an indication of the relative binding strength of the detergent to the protein and eventual stabilization of specific conformations. We also investigated the binding stoichiometry and conformational changes of α-syn monomers in the presence of both Ca^2+^ and specific detergents, since Ca^2+^ is known to be an important structure-modulating interactor. Here we sought to detect if Ca^2+^ binding influences detergent binding, or vice versa, and distinguish additional conformational effects when both detergent and Ca^2+^ are present as is the case in vivo.

## 2. Results

### 2.1. Interaction of Detergents with α-Synuclein

**Figure 1:**
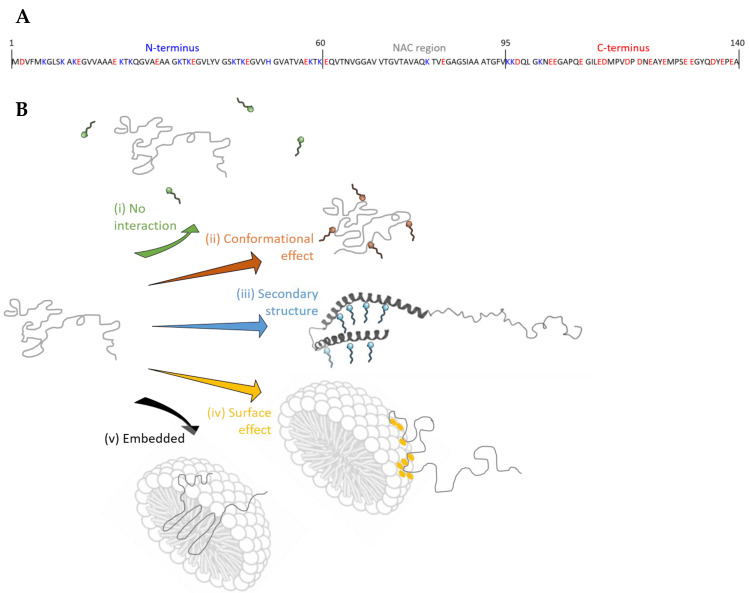
(**A**) Amino acid sequence of human wild-type α-syn. Negatively charged residues are in red, positively charged residues are in blue. The three different regions which can be distinguished are indicated. (**B**) Different scenarios that can occur for protein-detergent interactions. It can be that (i) no interaction is detected; (ii) that the tertiary protein conformations are altered; (iii) that secondary structure forms; (iv) that proteins interact with the surface of micelles; (v) or that they are embedded in the micelle.

When detergents interact with α-syn (amino acid sequence shown in Figure 1A), a number of different scenarios may occur (Figure 1B) depending on the detergent concentration: (i) no detergent interactions are detected (no binding, or binding not retained in the gas phase, see below); (ii) protein monomers retain a number of detergents bound that are detected in the mass spectra, possibly with accompanying conformational effects at the level of tertiary protein structure (overall compaction or extension); (iii) or detergent binding can cause changes to the secondary protein structure. Below the CMC, detergents are present as individual molecules, while all additional detergent above the CMC forms micelles with a concentration equal to the CMC that remain as individual molecules in solution [32,34,35]. In the presence of detergent micelles (above the CMC), there is the possibility that (iv) the protein may bind peripherally to the surface of detergent micelles, released by gentle collisional activation and detected with or without detergent still attached. The protein could also be embedded in micelles (v), but its release and detection would then require more substantial collisional activation in the mass spectrometer. In all these cases, if detergent binding is too weak or too hydrophobic to be retained inside the mass spectrometer, conformational “memory” effects of interaction may still be detected using charge state distributions or ion mobility. As the number of positive charges (protonations) which the protein acquires in ESI reflect the extent of exposed surface, with low charge states representing more compact conformations and high charge states more extended states, changes in the charge state distribution indicate conformational changes of the protein.

From our observations here it is unlikely that α-syn is embedded in micelles, as option (v) suggested. When mass spectra were acquired, there was no indication that the protein needed to be released from the micelle first, using more energetic conditions, and no typical micelle signals at higher *m*/*z* ranges were detected. This leaves us with four important possible modes of α-syn-detergent interactions (i–iv).

We tested selected detergents for binding to monomeric α-syn and, as a reference, to β-lactoglobulin (β-lac) to investigate which detergents are able to bind to α-syn monomers and their stoichiometry, which indicates the binding capacity of the protein per detergent. Using identical low energetic conditions in the mass spectrometer for all samples, we attempted to keep protein-detergent interactions intact throughout the measurement process.

### 2.2. Detergent Binding Capacity and Stoichiometry

**Figure 2:**
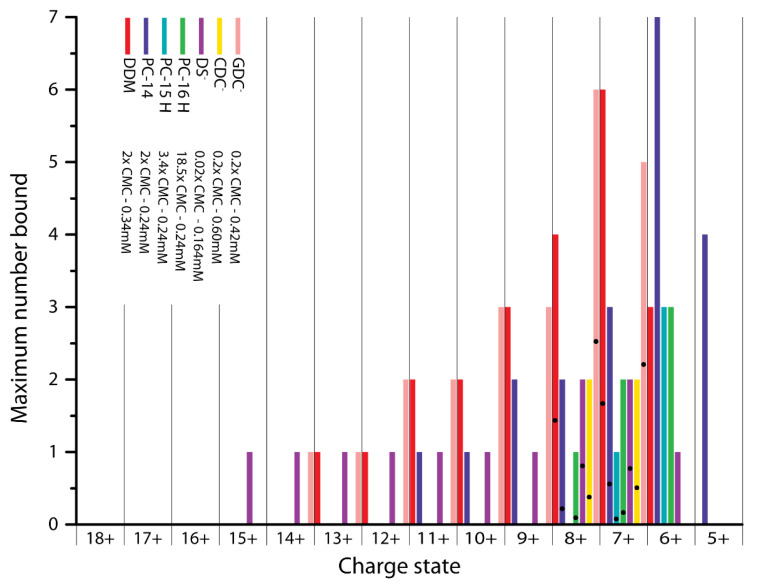
Comparison of the number of detergent molecules bound per charge state per detergent (DDM 2x CMC, PC-14 2× CMC, PC-15 3.4× CMC, PC-16 18.5× CMC, SDS 0.02× CMC, SCDC 0.2× CMC and SGDC 0.2× CMC). PC-15, PC-16 and CDC^-^ only bind to lower (compact) charge states while DDM, PC-14, DS^-^ and GDC^-^ also interact with higher (extended) charge states. The average number of molecules bound per detergent for the 7+ and 8+ charge state is indicated by dots.

Most published studies of the membrane binding capacity of α-syn focus on negatively charged membrane components, more specifically on SDS when it comes to detergents, as it is known that the positively charged N-terminal region of the protein interacts with these negative charges [5,6,36,37]. Besides the ionic charge of a detergent, its degree of hydrophobicity plays an important role for its behavior in solution as well as interactions with proteins [38]. Our results clearly show that α-syn does not only bind to detergents or lipids with a negative head group, but that binding to different types of detergents occurs (Figure 2). Here n-dodecyl β-D-maltoside (DDM) and n-tetradecylphosphocholine (PC-14) are present at a concentration of 2× CMC. For n-pentadecylphosphocholine (PC-15) and n-hexadecylphosphocholine (PC-16) the final detergent concentration was the same as for PC-14, 0.24 mM, equivalent to 3.4× CMC for PC-15 and 18.5× CMC for PC-16, respectively (indicated with an ‘H’ in Figure 2). Sodium dodecyl sulfate (SDS) was used at 0.02× CMC and both sodium chenodeoxycholate (SCDC) and sodium glycodeoxycholate (SGDC) were present with a final concentration of 0.2× CMC as the quality of the data was not good with concentrations above the CMC. For comparison, binding stoichiometries for the interaction of detergents with β-lac are shown in Supplementary Figure S1.

α-syn has a high binding capacity for the non-ionic detergent DDM, with a maximum of six DDM molecules bound to charge state 7+. For the other non-ionic detergent tested, Triton X-100, the intensity of the signal of the bound states is however very low compared to DDM (Supplementary Figure S2). DDM has a bulky hydrophilic group and a long hydrophobic chain, and it is known that hydrophobic interactions play an important role when binding to proteins [39]. In addition, the presence of the sugar moiety enables DDM to form hydrogen bonds with the protein, while this option is limited for Triton X-100. It has been shown before that Triton X-100 interactions are hard to detect using MS due to their hydrophobic nature, as micelles tend to fall apart easily and only little protein-detergent binding is retained [40]. Due to the low intensity of Triton X-100 adducts, this detergent was excluded for the rest of the study.

Zwitterionic detergents (PC-14, PC-15 and PC-16) also bind to α-syn, and the binding capacity is significantly higher for PC-14 with up to seven molecules bound to the 6+ charge state compared to a maximum of three PC-15 or PC-16, at the same concentration. This indicates an effect of the chain length on the binding capacity and possibly affinity (see further discussion below).

For SDS, SCDC and SGDC, only binding of the detergent anions without inclusion of the Na^+^ counterion is detected; labelled here as DS^-^, CDC^-^ and GDC^-^ respectively. It is expected that DS^-^ binds well as it was previously reported that α-syn interacts with SDS micelles [5,6,36,37]. SDS is commonly used as a denaturant for polyacrylamide gel electrophoresis where it binds proteins in a 1.4:1 *w*/*w* ratio, so roughly one SDS molecule per two amino acids [35]. Somewhat surprisingly though, with cetrimonium bromide (CTAB) only binding of Br^-^ can be observed (Supplementary Figure S3) but no interaction with the detergent cation. Therefore this detergent was also excluded from the study. For α-syn with its high, negative net charge at neutral pH, this lack of interaction with the cationic detergent is somewhat unexpected. CTAB lacks the phosphodiester group of PC-14 but has an otherwise identical structure (see method section), indicating that this negative charge, and the possibility to form additional hydrogen bonds, must play a key role. A previous study with bovine serum albumin detected binding sites close to aspartate or glutamate residues for cationic detergents. An approximately 10× higher concentration was however needed to achieve similar binding as with anionic detergent [41].

### 2.3. Conformational Selectivity of Detergent Binding

Besides the overall binding capacity, we are interested in how detergents bind to different charge states of the α-syn monomer. This can give insight if specific detergents have a prevalence for binding to, or shifting the equilibrium towards more compact or extended conformational states, since the number of charges a protein picks up during the ESI process is directly related to its conformation and more specifically its solvent accessible surface area (SASA). Low charge states represent more compact conformations and high charge states indicate more extended states (see method section). α-syn as a natively unfolded protein displays a broad range of charge states from 5+ to 18+, forming a multimodal distribution.

As seen in Figure 2, PC-15, PC-16 and CDC^-^ only bind to lower charge states (8+ to 6+) while DDM, PC-14, DS^-^ and GDC^-^ can bind to higher charge states as well (up to 13+, 11+, 15+ and 14+ respectively). This indicates that detergent binding is conformation specific, where PC-15, PC-16 and CDC^-^ selectively bind to more compact (low charge) states while the other detergents seem to be indiscriminate.

For charge states 7+ and 8+ the average number of detergent molecules bound, based on the weighted average of all stoichiometries observed including the unbound state, is indicated in Figure 2 with a black dot. Both charge states represent the compact conformations of α-syn and the average detergent number is comparable between them. The series of detergent adduct peaks in the mass spectra, as shown in the method section for DDM adducts and representative for all detergent binding to α-syn, shows a smooth and gradual decrease in intensity, indicating that there are no preferred binding stoichiometries for any of the detergents tested.

While we can see that binding of some detergents is conformation-specific (i.e., charge state related), the question arises if detergent binding can remodel the structure and thereby shift the charge state distribution of the protein. While the charge distribution is not altered when detergents are present, intensity shifts for the lower charge states can be detected for different charge states (Supplementary Figure S4). Eventual denaturing effects (as described for e.g., SDS) caused by specific detergents might also play a role in the binding stoichiometry as they can unfold compact conformations, leaving the charged backbone more exposed leading to more possible ionic interactions and a higher detergent stoichiometry. This effect will however be limited for α-syn as it is natively unfolded and already occurs in more extended conformations. Conformational effects of binding can be analyzed in much finer detail using ion mobility and are further discussed in later sections.

Intensities of α-syn dimer peaks, also present in the spectra (Supplementary Figure S3), do not change significantly with detergent present. Under the conditions studied here, we therefore do not detect any significant effects of detergent on the oligomerization of α-syn.

### 2.4. Conformational Effects of Detergent Binding

As we had established to what extent detergents interact with compact and extended states of α-syn, we next assessed the key question of how the conformational ensemble responds to the presence of detergent, using IM-MS.

**Figure 3:**
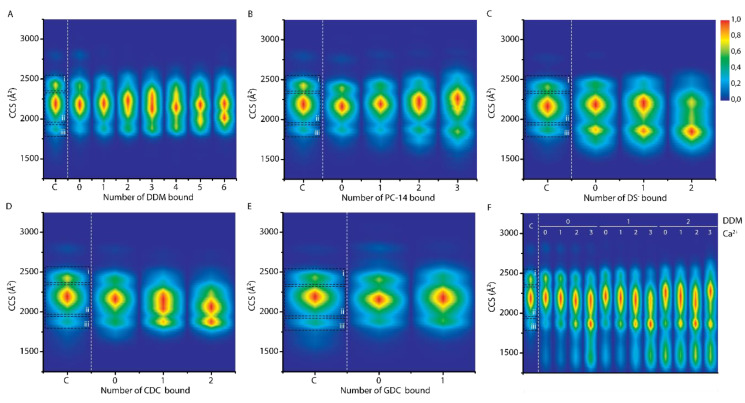
(**A**–**E**) CCS plots showing the conformational effects per additional detergent molecule interacting with the 7+ charge state of α-syn. On the x-axis “C” indicates the control, the unbound state of the 7+ charge state of α-syn without detergent present. “0” indicates the unbound state of the 7+ charge state of α-syn with detergent present (detergent concentrations used: 2× CMC DDM and PC-14, 0.02× CMC SDS, 0.2× CMC SCDC and SGDC). (**F**) CCS plot when 2× CMC DDM and 400 µM CaCl_2_ is present. Intensities are scaled as described in the method section.

In Figure 3 CCS plots are shown for all detected detergent stoichiometries at the 7+ charge state of α-syn (A: DDM, B: PC-14, C: DS^-^, D: CDC^-^, E: GDC^-^). Comparison of the CCS plots of PC-14, PC-15 and PC-16 is discussed later. In these plots “C” indicates the control, i.e., the unbound state without detergent present, “0” indicates the unbound state but with detergent present in the sample, and higher numbers indicate the number of detergent molecules bound as identified by the additional mass. We would expect that the CCS profiles in the presence of detergent (“0”) mirror exactly the situation without detergent present (“C”), but in cases where detergents bind weakly in solution and are lost in the gas phase of the mass spectrometer, a conformational memory, i.e., CCS contributions from lost bound states, may be seen. This is clearly the case here for DS^-^ binding where the “0” profile resembles more the one-detergent bound state than the control. In general three conformational families can be distinguished by CCS for the control 7+ charge state, indicated in the control lane, with conformation “i” being the most extended one and conformation “iii” being the most compact one.

With increased DDM binding (Figure 3, panel A), a slight, gradual shift of the CCS profiles towards higher values is detected. We interpret this as the small size increase which comes with the additional volume of bound detergents, rather than a conformational shift of the protein itself [42]. The most important trend observed here as such is the formation of more compact conformations within conformational family “ii” when three and more DDM molecules bind to α-syn. An additional conformation which lies in between conformations “ii” and “iii” also appears. This compacting effect of DDM for the 7+ charge state runs in parallel with the general intensity shift towards more compact conformations observed in the charge state distribution (Supplementary Figure S4).

Figure 3, panel B depicts the CCS plot for the zwitterionic detergent PC-14. Besides a similar slight shift towards higher CCS values, from the second detergent onwards again more compact conformations within conformational family “ii” occur. This indicates that the net charge of a detergent molecule alone, zero for both DDM and PC-14, does not define the structural effect it has on α-syn and that different modes, and likely also sites, of interaction play an important role in how conformations are altered.

For DS^-^ there was an intensity shift towards conformational family “iii”, distinct from the effects seen for DDM and PC-14. Binding of CDC^-^ results in a shift within conformational family “ii” as well as an increase in intensity of conformational family “iii”, resulting in a different and unique pattern. For GDC^-^ however there is only a very slight change observed which we consider insignificant. This indicates that the difference in structure between CDC^-^ and GDC^-^, which amounts to an additional amide group and an altered position of one of the hydroxyl groups, results in significantly different interactions and structural effects. This is in spite of the similar structure and same net charge of the detergent, but most likely due to an alternative hydrogen bonding pattern. Based on the intensity shifts towards higher charge states, as seen in Supplementary Figure S4, we propose that these anionic detergents have similar denaturing effects as SDS [34]. Upon binding to specific lower charge states, however, a compacting effect could be detected. Comparison of these CCS plots exemplifies the importance and the detail with which conformational ensembles can be detected with IM measurements.

Earlier studies have suggested that DDM can bind to proteins in different ways, either by hydrophobic interactions with its aliphatic tail, or via hydrogen bonds with its hydrophilic sugar moiety [39]. We can assume that if a non-ionic detergent such as DDM can interact with a protein with its hydrophobic part, charged detergents with similar structured hydrophobic groups, such as fos-choline derivatives, should be capable of this as well. Furthermore, it is reasonable to assume that charged head groups can interact with some of the many charged side chains of α-syn via electrostatic interactions, as well as forming hydrogen bonds. As an example, molecular dynamics (MD) simulations in water indicated that the interaction between DDM and hydrophobic residues can be stronger than its interaction with hydrophilic residues and charged groups [39]. The hydrophilic head group of DDM can, in theory, form a high number of hydrogen bonds. However, the coordination between the protein and the detergent must be very specific for more than one hydrogen bond to form with the bulky hydrophilic head group, increasing the importance of hydrophobic interactions.

It has further been proposed that, besides strong hydrophobic interactions, the basic lysine residues might play an important role in the interaction of proteins with the negatively charged surface of SDS micelles [34,43]. α-syn contains fifteen lysine residues in its amino acid sequence, most of them in the N-terminal part. If the positive charges of these residues are (partly) neutralized by DS^-^ binding, long-range electrostatic effects that shape the overall protein structure can be altered. Considering that α-syn is already largely unstructured in solution, it is expected that changes in local, and global, charge density will affect the local, and global, conformational behavior of the protein, in this case resulting in more compact protein conformations.

For DS^-^ binding there already seems to be a compacting effect for the unbound state with SDS just present in solution (Figure 3C). This indicates a memory effect of bound states, and that at least some DS^-^ interactions are lost during the analytical process. For CDC^-^ binding this effect is less significant and for GDC^-^ it is absent. Ionic detergents are more likely to induce denaturation of the protein, apart from bile acid derivatives such as SCDC and SGDC that are more mild in this respect, than zwitterionic and non-ionic detergents [44]. Nevertheless, we do not see any shifts in the range of charge states or increases in CCS value that suggest full denaturation of the protein (i.e., complete loss of the compact states). Even very similar compounds such as CDC^-^ and GDC^-^ result in different interactions and conformational patterns, reinforcing the hypothesis that individual detergent molecules have quite specific effects.

In Supplementary Figures S5 and S6 analogous CCS plots can be seen for the 6+ and the 8+ charge states, indicating that the conformational trends are not charge state specific within the group of compact states. In comparison, Supplementary Figure S7 represents the CCS plot for the 11+ charge state of α-syn with DDM bound, indicating that for higher charge states representing more extended conformations, charge repulsion is more difficult to overcome and no conformational effects are detected apart from the gradual increase in CCS values due to the additional volume of DDM molecules.

In Supplementary Figure S8 CCS plots of β-lac in the presence of specific detergents are shown. No conformational changes could be detected, indicating that conformational effects we see for α-syn are specific for this protein, and that globular and folded proteins such as β-lac are less susceptible to conformational effects as a result of detergent interaction.

Since in vivo, metal ions such as Ca^2+^ are active at biological membranes and known to play a role in remodeling the conformation of α-syn so it can direct vesicle release, the interaction of Ca^2+^ and detergents with α-syn was also investigated. Ca^2+^ binding is known to result in compaction of the protein structure when binding to α-syn [45,46]. Mass spectra confirmed that detergent binding does not have an effect on the number of Ca^2+^ ions that can bind to the protein (data not shown). Conversely it is more difficult to investigate whether Ca^2+^ binding has an effect of the detergent binding stoichiometry, since noise levels are significantly increased. For most detergents it however does not seem as if there is a large shift in binding stoichiometries with and without Ca^2+^ present, only for DDM the number of detergents bound seems to be decreased. In panel F of Figure 3, the CCS plot of the 7+ charge state of α-syn is shown when both CaCl_2_ and DDM are present in the sample (400 µM and 2× CMC, respectively) and able to interact with the protein. A cumulative compacting effect is observed when both DDM and Ca^2+^ bind to α-syn, increasing when more Ca^2+^ ions bind at a certain detergent stoichiometry. Additional very compact conformations occur as well, which were not present when only DDM or only Ca^2+^ was bound to α-syn. This indicates that the interaction between α-syn, detergents and Ca^2+^ can impact protein structure profoundly. Supplementary Figure S9 shows a control CCS plot of α-syn with only CaCl_2_ present in a 1:20 protein to metal ion ratio, and a CCS plot with both CaCl_2_ and PC-14 or SCDC present.

### 2.5. Probing Conformational Transitions

**Figure 4:**
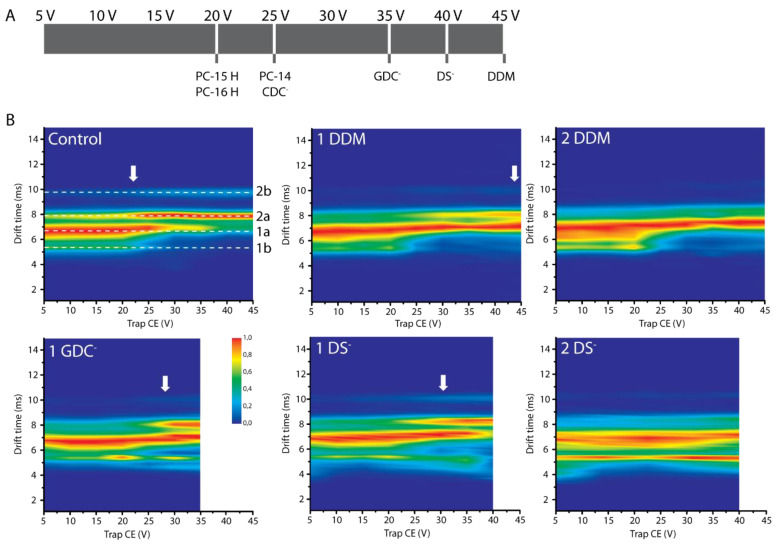
Conformational transitions probed by CIU. (**A**) The lines indicate at which trap CE voltage binding of different detergents to the 7+ charge state of α-syn is lost (no binding: S/N from peak with 1 detergent bound ≤3). (**B**) CIU plots of the 7+ charge state of α-syn. Top row: control without detergent present, with one DDM and with two DDM bound. Bottom row: with one GDC^-^ bound, and one and two DS^-^ bound. Intensities are scaled as described in the method section. Four different conformational families are labelled 1a, 1b, 2a and 2b in the control plot. Here ‘1’ indicates more compact states, ‘2’ indicates more extended conformations, ‘a’ indicates more intense states and ‘b’ indicates less intense conformations. The CIU50 value, indicating the transition between conformation 1a and 2a, is indicated by the white arrow in cases where unfolding occurs before detergent binding is lost. When detergent binding is lost below 45 V, this is indicated by blank spaces in the plots.

Our initial workflows employed gentle energetic experimental conditions to retain the protein in its native state, and detergent interactions intact, during the course of the experiment. When using gradually more energetic and activating conditions, by increasing acceleration voltages in the instrument, information related to the relative binding strength of different detergents can be obtained as well as eventual stabilizing or destabilizing effects of bound detergent for specific conformations. In these CIU plots, increasing the trap collision energy in steps of 5 V will eventually result in the loss of detergent interaction, generally indicated by the CID50 value when 50% of the ligand is lost, usually concomitant with protein unfolding [47]. The more activation needed to break the interaction, the stronger the binding. In Figure 4A the acceleration voltages are shown when most detergent binding to the 7+ charge state of α-syn is lost.

CIU plots of 7+ α-syn without detergents present, as a control, and with various detergents bound demonstrate their stabilizing effects on certain protein conformations (Figure 4B). For the control we see two prominent states (at drift times of 6.61 and 7.85 ms, labelled 1a and 2a respectively) with a CIU50 value, the midpoint of the transition between the two features, of 22.1 V, indicated by a white arrow. Supplementary Figure S10 shows how we determined the CIU50 value, while Gaussian fitting of the drift time plots before (20 V) and after the transition (25 V) clearly show two additional, less intense conformations, a very compact one and a very extended one, labelled 1b and 2b respectively, representing the structural diversity of α-syn. Using the control plot as a reference, stabilizing or destabilizing detergent effects on these conformations can be defined. Only detergents with CIU50 values above 25 V are shown as the stabilizing effect is otherwise considered too insignificant.

When one or two DDM molecules are bound, the compact conformation 1a persists until at least 45 V, while for higher voltages most of the binding is lost (cf. Figure 4A). The transition to the more extended conformation 2a only appears to begin around 40 V with one DDM molecule bound, and not yet with two. As DDM is non-ionic, no global long-range electrostatic effects but only local interactions can play a role here. This means that hydrophobic interactions and hydrogen bond formation must result in this relatively strong interaction. The anionic detergent GDC^-^ has a stabilizing effect on compact conformations. Transition only appears at a voltage of 25 V and higher. This is comparable to when one DS^-^ is bound, where conformation 1a is again stabilized until voltages of 35 V while transition to more extended states occurs when voltages of 25 V and higher are applied. When two DS^-^ ions bind, both compact conformations are stabilized and no transition to 2a occurs before binding is lost. However, CDC^-^ only remains bound up to 25 V. The main difference between SCDC and SGDC is the number of hydrogen bonds that can be formed, with an additional amide group in SGDC. Due to the longer chain carrying the negative charge in SGDC, more and tighter interactions may be possible which would explain the stronger binding seen in this case. Steric hindrance may also prevent tighter binding for SCDC, compared to SDS and SGDC.

We have previously shown that the interaction with DDM, DS^-^ and GDC^-^ results in apparent compaction of α-syn and it seems these detergents can as well stabilize these more compact forms. This demonstrates that different types of detergents can in general have stabilizing or destabilizing effects on certain protein conformations, yet the final CIU pattern is specific per detergent indicating specific ways of interaction for each detergent. Furthermore, stabilizing effects of detergents seem to be additive, i.e., each additional detergent molecule shifts the unfolding transition further. When one detergent molecule is bound, the stabilized and more compact conformation 1a is still present at the highest voltage where binding of the detergent is observed. This indicates that the protein-detergent interaction is disrupted before conformational change occurs. In Supplementary Figure S11 analogous CIU plots of the 8+ charge state with detergents are shown.

### 2.6. Detergent Concentration Effects

**Figure 5:**
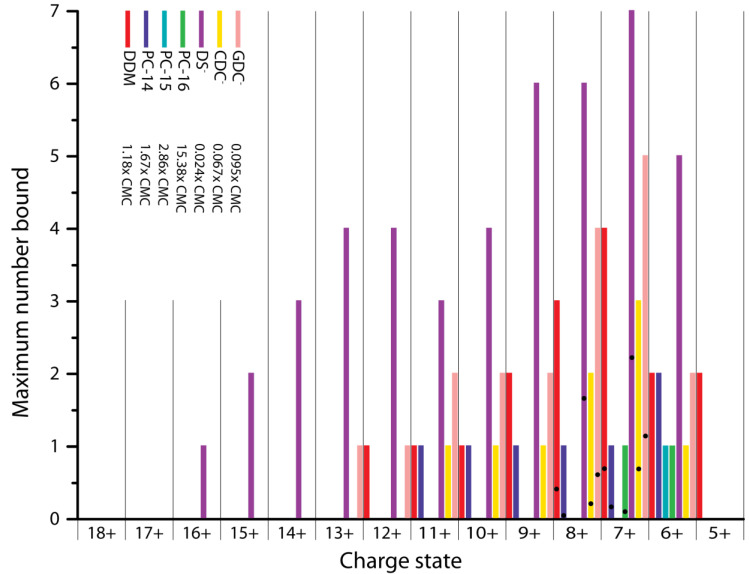
Comparison of the number of detergents bound per charge state and detergent when the detergent concentration is fixed at 0.20 mM (1:20 protein:detergent ratio).

To investigate if the detergent concentration, and importantly the presence of micelles (above CMC), have an effect on the interactions with α-syn, we also performed experiments at a different, fixed concentration of 0.2 mM of each detergent. Interconversions between× CMC and mM for all detergent concentrations used are shown in Table 1. In Figure 5, similar to Figure 2, the maximum number of detergents bound per charge state is shown at a detergent concentration of 0.2 mM.

For DDM, PC-14, PC-15 and PC-16, indicated in yellow in Table 1, the detergent concentration always remains above the CMC; hence the concentration of free detergent should remain unchanged while the micelle concentration decreases compared to when the “standard” concentrations are present. These four zwitterionic detergents show lower binding stoichiometries at 0.2 mM than what was seen in Figure 2 (Table 1, PC-15 H and PC-16 H). This indicates that the micelle concentration plays a role, and there must be an interaction with the micelles rather than just with individual detergent molecules. We detect a number of detergents from these micelles which remain attached to the protein as described in situation iv, Figure 1.

Anionic detergents were used below the CMC, as otherwise no good signal could be obtained at the required high concentrations. Without micelles, the concentration corresponds to the amount of free detergent. The concentration of SCDC and SGDC is now lower at 0.2 mM, which is expected to result in lower binding stoichiometries. For CDC^-^ binding of up to three ions was detected and over a broader charge state range compared to Figure 2, where a maximum number of two ions was bound. For GDC^-^ the results are similar to Figure 2. This may indicate that α-syn has specific binding sites or regions for SCDC and SGDC so that a change in concentration does not result in a drastic change in binding stoichiometry. For SDS at a higher concentration of 0.024× CMC, compared to 0.02× CMC, we find that up to seven DS^-^ molecules can now bind to α-syn, a number that is significantly increased compared to what was seen in Figure 2. A high number of DS^-^ binding events are detected here for lower charge states as well as for higher charge states, indicating less conformational selectivity for DS^-^ binding.

**Figure 6:**
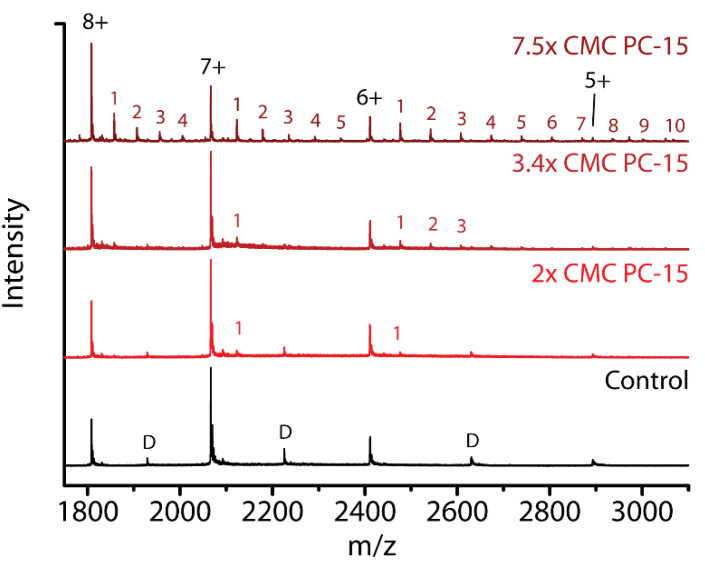
Mass spectra of the lower charge states when no detergent is present (Control) and when different concentrations of PC-15 are present (2× CMC, 3.4× CMC and 7.5× CMC). A significant increase in number of PC-15 molecules bound is detected when increasing the concentration from 3.4× CMC to 7.5× CMC. Dimer peaks are indicated with a ‘D’.

Figure 6 shows spectra of the compact charge states without any detergent (control) or with 2× CMC, 3.4× CMC and 7.5× CMC of PC-15 present. The stoichiometries detected for 2× and 3.4× CMC (cf. Figure 2) are very low with only one detergent molecule bound for the 7+ charge state. This number increases to up to five molecules bound when the concentration of PC-15 is increased to 7.5× CMC. This is similar to the increase in PC-14 binding when increasing the concentration from 0.2 mM to 0.24 mM (Figure 2 and Figure 6, respectively). This indicates that above the CMC, the interaction with higher concentrations of micelles must induce the increased stoichiometry of detergents bound. As the aggregation number for these detergents increases with increasing chain length from PC-14 to PC-16, the concentration of micelles decreases as the micelle concentration equals the detergent concentration minus the CMC, divided by the aggregation number [48]. This may suggest that with a higher micelle concentration, more detergents retain bound when the protein detaches from the micelle or when the micelle falls apart.

As the stoichiometry of binding depends on the concentration of the detergent, below as well as above the CMC, it is important to determine if interactions with free detergent and micelles influence the conformational behavior of the protein in a similar way. Supplementary Figure S12 compares CCS plots of DDM, PC-14, CDC^-^ and GDC^-^ binding to α-syn when the detergent concentration is either 2× CMC for DDM and PC-14 or 0.2× CMC for SCDC and SGDC, or is equal to 0.2 mM in all cases. No discernible concentration effect on the conformational pattern is detected for these detergents. In Supplementary Figure S13 the CCS plots are shown of α-syn at 0.02× CMC SDS and when 0.2 mM, or 0.024× CMC SDS, is present. As discussed before in Figure 3, there is however some crosstalk between the bound and unbound states because of the “memory effect”, which explains the apparent differences observed here. In summary our data suggest that there is no effect on the conformational pattern related to the presence and abundance of micelles in the sample, only the actual retained binding stoichiometry plays a role here.

### 2.7. Effect of Chain Length within the Same Detergent Class

**Figure 7:**
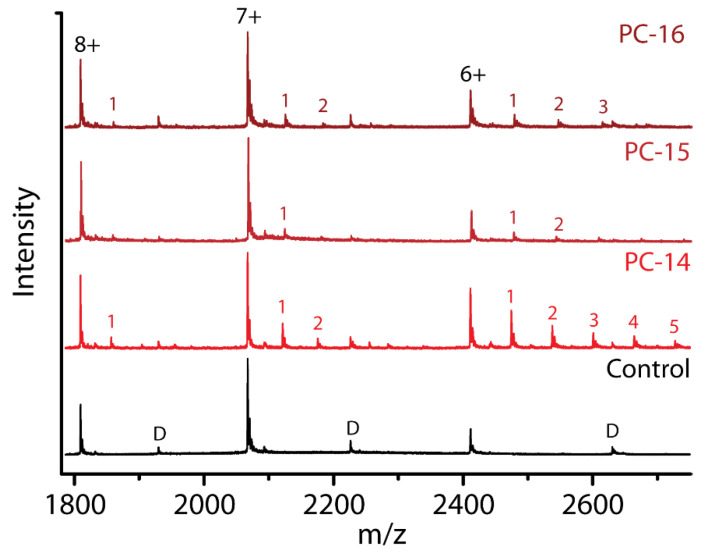
Comparison of fos-choline binding to α-syn. Control, without detergents present, and with PC-14, PC-15 or PC-16 at a concentration of 0.24 mM (2× CMC PC-14, 3.4× CMC PC-15 and 18.5× CMC PC-16)). Dimer peaks are indicated with a ‘D’. Numbers indicate binding stoichiometries of the respective detergent.

Previous studies have reported the curvature of the bilayer as an important factor in the interaction between α-syn and biological membranes [19]. The curvature of micelles depends on the shape and (chain) length of the detergents. For the fos-choline detergents, the micelle size and packing density increases with increasing chain length from PC-14 to PC-16, resulting in lower curvature of the micelle surface [49]. We queried whether there was such an effect on the binding stoichiometry and conformations of α-syn in interaction with micelles (scenario iv of Figure 1B). In Figure 7 mass spectra are compared at a fixed detergent concentration of 0.24 mM in the sample.

α-syn clearly retains the most detergent molecules for PC-14, indicating that it might have a preference for interacting with PC-14 micelles with their higher curvature and packing density, whereas the headgroups are identical for all fos-cholines. The protein likely interacts with a larger part of the PC-14 micelle surface or interacts more strongly. As packing density increases with increasing chain length, the difference in stoichiometry may also be explained by how easily detergent molecules are detached from the micelle by the protein and how easily micelles can fall apart. When the packing density is lower, detergent molecules might be more easily removed from the micelle and the micelle structure will be less stable and fall apart more easily [50].

Supplementary Figure S14 visualizes how intensities for the compact form of α-syn (7+ and 8+ charge states) change when PC-14, PC-15 and PC-16 are present (2x CMC as well as 0.24 mM final concentration). In all cases there seems to be a decrease in intensity; i.e., a shift towards the more extended forms of the protein.

**Figure 8:**
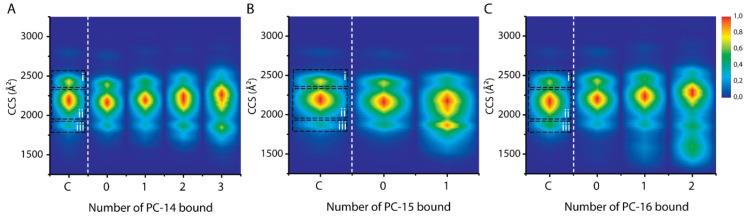
CCS plots of different binding stoichiometries for PC-14, PC-15 and PC-16 present with a concentration of 0.24 mM (2x CMC PC-14). (**A**) is identical to (**B**) of Figure 3. Conformational families “i” (more extended) to “iii” (more compact) are indicated in every sub-figure in the dashed boxes. (**C**) on the x-axis indicates the control, the unbound state of the 7+ charge state of α-syn without detergent present. “0” indicates the unbound state of the 7+ charge state of α-syn with detergent present. Intensities are scaled as described in the method section.

In Figure 8, CCS plots show conformations of the 7+ charge state of α-syn with PC-14, PC-15 and PC-16 at equal concentrations of 0.24 mM.

While we saw that the binding affinity of PC-14, PC-15 and PC-16 is quite different depending on the chain length, the structural effects are comparable. This indicates that the detergent affinity is determined by the micelles and their properties that differ due to the hydrophobic chain length, while interaction sites with the protein are probably determined by the polar headgroup and its capacity to form hydrogen bonds and other electrostatic interactions, which are shared between these zwitterionic detergents. This again supports the hypothesis that, maybe in addition to interaction with individual detergent molecules, there must be specific interactions with micelles.

## 3. Discussion

As α-syn accounts for approximately 1% of the cytosolic proteins in neuronal cells, the in vivo concentration can be up to 100 µM [1,51]. These concentrations can fluctuate and also differ dramatically according to the location in the human body where α-syn is found. Interactions with other proteins, organelles, and of course lipids in biological membranes also determine the effective local concentration of the protein inside the cell. For detailed molecular studies, it is therefore currently unrealistic to reproduce these diverse in vivo scenarios in biophysical experiments. In our nESI-IM-MS experiments, we used various different detergents both above and below the CMC to study interactions and structural effects with α-syn. A protein concentration of 10 µM was chosen to avoid aggregation and obtain good signal with little noise. The interaction between α-syn and biological membranes has gained a lot of interest recently, and we are using amphiphilic compounds as models for lipids in order to contribute an increased understanding how biological membranes can affect α-syn structure, and possibly affect biological function as well as determining the aggregation pathway. An earlier study concerning IAPP, an amyloidogenic peptide involved in diabetes, showed that insertion of this peptide into biological membranes is driven by free lipids [52]. This also motivated the present study which investigates the interaction of α-syn with individual detergent molecules as well as micelles.

Considerable research has focused upon biological and biophysical characterization of the lipid composition of neuronal membranes. In spite of lacking consensus, studies have shown that membranes of synaptic vesicles are mainly composed of phosphatidylcholine, phosphatidylethanolamine and cholesterol [28,29,30]. The former two are zwitterionic lipids whose interactions might be mimicked by the zwitterionic detergents used in this study, whereas cholesterol shares a similar backbone structure as the bile salt derivatives used here. Phosphatidylserine is another relatively abundant lipid in the cell membrane, and with its negatively charged headgroup it contributes to the overall negative charge of biological membranes [28,30]. We selected anionic detergents that share structural similarities, while not able to exactly mimic components of biological membranes in our experiments. Using a relatively simplistic membrane model, our data provides an important and valuable first step to study protein-membrane interactions at the molecular level.

We have established that different types of detergents can interact with the protein α-syn, and that this interaction does not only depend on the overall charge of the detergent, but that the structure of both the hydrophilic and hydrophobic domain, and the contributions of different types of interactions (hydrophobic, hydrogen bonds and electrostatic) of a detergent play a very important role. This results in diverse binding stoichiometries, indicating different binding capacities of α-syn per detergent. We saw that the binding stoichiometry depends on the type of detergent and the concentration, with clear effects both below and above the CMC. We also saw that most detergents preferentially bind to more compact, lower charge states. DDM, PC-14, DS^-^ and GDC^-^ can however also be found interacting with higher charge states.

A key aspect of this study was to see if detergents would alter the conformational space of α-syn monomers in a specific way. nESI-IM-MS experiments prove that most types of detergents do not only prefer binding to more compact conformations, but also have a common compacting and stabilizing effect. When looking more closely however we see unique conformational footprints for each detergent indicating more specific conformational effects, caused by specific interaction modes and most likely also differences in binding sites. In addition, we assessed how the interaction of a specific detergent in combination with Ca^2+^ was able to alter the conformational space of the protein or affect detergent binding. A cumulative compacting effect was observed, again different in detail for every detergent tested. This confirms recent studies that Ca^2+^ is a very important factor in the interaction between α-syn and biological membranes and that it can cause important structural changes [21].

There are specific regions of α-syn that are known to be important for interacting with membrane mimicking environments, such as the positively charged N-terminus which can form an amphipathic α-helix when binding to negatively charged vesicles [18]. Helix formation can also be induced by zwitterionic headgroups of the detergent. These helical structures are different from the helices formed when α-syn binds to SDS micelles [53]. Hydrophobic regions of both detergents and the protein, in the case of α-syn the NAC-region, are also important. Some detergents, e.g., DDM, are known to interact very strongly with a protein due to hydrophobic interactions, likely in addition to polar interactions [39]. As significant differences in stoichiometry and binding affinity were found between the structurally related bile acid derivatives SCDC and SGDC, the formation of specific hydrogen bonds must play an important role. We were however surprised not to detect any binding of the cationic detergent CTAB. It has the same hydrophobic tail as PC-14, for which we see strong α-syn binding, and a headgroup that may well be expected to interact with the many negative charges on the C-terminus of the protein. This example highlights a need for further investigation of what exactly is needed to establish a detergent interaction with α-syn, but also with proteins more generally.

Different modes of protein-detergent interaction were suggested in Figure 1B, and based on our data it is clear that both individual detergent and micelle/surface interactions occur. As there was no binding observed with cationic CTAB, this would fall under option (i). For DS^-^ there are clear indications that at least some of the interactions were lost during the experiment, however detergent binding was still detected. Through binding of detergent molecules below CMC concentrations, and free detergent and micelles present above the CMC, the formation of secondary structures is expected for SDS according to the literature (option (ii)). Ion mobility measurements detect the global, overall size and shape of a protein and are therefore not expected to be sensitive to changes in the secondary structure alone. Where the tertiary protein structure (overall compactness) changes, ion mobility can register this as suggested in option (iii) (Figure 1B) and evidenced in Figure 3. We also find, by comparing different detergent concentrations, that interactions with micelles occur as described in option (iv), and that these interactions depend on the nature of the detergent and the properties of the micelles such as its curvature.

There are many different possibilities for how α-syn can interact with a biological membrane, and there are still many questions on what happens at the molecular level [32], including what roles different isoforms play. α-syn in vivo carries post translational modifications (PTMs) and it is known that these can have a drastic effect on the interaction with (charged) structural modulators and protein conformations [54]. The interaction between biologically relevant α-syn proteoforms e.g., N-acetylated or phosphorylated isoforms, detergents and biological membranes therefore represents a vital subject for further investigations.

## 4. Materials and Methods

Materials and sample preparation: β-lactoglobulin (β-lac) from bovine milk (Sigma Aldrich, St. Louis, MO, USA) was dissolved in a 20 mM ammonium acetate (Sigma-Aldrich, St. Louis, MO, USA) pH 7 solution with a final protein concentration of 10 µM. Human wild-type α-syn was expressed in *E. coli* Bl21(DE3) in a pT7-7 based expression system and purified using the method previously described [55]. Before anion exchange chromatography, an additional gel filtration step was implemented for additional purification. The purified protein was dialyzed against 20 mM ammonium bicarbonate (Sigma Aldrich, St. Louis, MO, USA) and lyophilized. Lyophilized α-syn was dissolved in aqueous 20 mM ammonium acetate pH 7 with a final stock concentration of 70 µM. The experimental mass is 14,461.77 ± 1.14 Da, while the theoretical average mass of the protein is 14,460.1 Da. n-Dodecyl β-D-maltoside (DDM), n-tetradecylphosphocholine (fos-choline-14 or PC-14), n-pentadecylphosphocholine (fos-choline-15 or PC-15), n-hexadecylphosphocholine (fos-choline-16 or PC-16) were purchased from Avanti Polar Lipids Inc. (Croda international, Snaith, UK), Triton X-100, cetrimonium bromide (CTAB), sodium dodecyl sulfate (SDS), sodium chenodeoxycholate (SCDC) and sodium glycodeoxycholate (SGDC) were purchased from Sigma Aldrich (Sigma Aldrich, St. Louis, MO, USA). Detergents were dissolved in deionized H_2_O with final stock concentrations of 50× CMC for DDM, Triton X-100, PC-14, PC-15, PC-16, 4× CMC for CTAB and SDS, and 2× CMC for the bile acid derivatives SCDC and SGDC, depending on the solubility. In the final sample the α-syn concentration was 10 µM in the presence of 2× CMC of DDM, Triton X-100, PC-14, PC-15, PC-16; 0.2× CMC of CTAB, SCDC and SGDC, and 0.02× CMC of SDS detergent. When other concentrations are used they are mentioned in the text. Samples were incubated at room temperature for at least 30 min after detergent addition before starting the measurements. In cases where detergent concentrations below the CMC were used, higher concentrations led to suppression of the protein signal and high noise levels, making acquisition of good quality MS data impossible. For PC-15 and PC-16, additional measurements were performed using concentrations equal to 2× CMC of PC-14; these measurements are referred to as experiments with ‘high concentrations’ of PC-15 and PC-16 present. Measurements were also performed for samples with equal detergent concentration of 0.20 mM while the concentration of α-syn remained constant at 10 µM. To investigate the effects of Ca^2+^ in combination with detergents, 400 µM CaCl_2_ was added to samples with a final α-syn concentration of 10 µM and 2× CMC DDM or PC-14, or 0.2× CMC SCDC. Table 2 shows the structure of the detergents used, arranged by type, with indication of their CMC value, molecular mass and the standard sample concentration.

Native nano-electrospray ionization and ion mobility-mass spectrometry: Measurements were performed on a Synapt G2 HD mass spectrometer (Waters Corporation, Wilmslow, UK) using a nESI source and borosilicate capillaries, pulled and gold coated in-house. The most important experimental parameters were set at: capillary voltage 1.4–1.7 kV; sample cone 40 V; extraction cone 1 V; trap collision energy 5 V; transfer collision energy 0 V; trap bias 40 V; IM wave velocity 300 m/s; IM wave height 35 V. Gas pressures in the instrument were: source 2.71 mbar; trap cell 0.024 mbar; IM cell 3.0 mbar; transfer cell 0.025 mbar.

Collision induced unfolding: In addition to native MS experiments, Collision Induced Unfolding (CIU) experiments were conducted [47]. For these experiments the same settings were used as for the native experiments, but the trap collision energy, the acceleration voltage into the trap cell of the instrument, was increased in a stepwise manner in 5 V increments.

**Figure 9:**
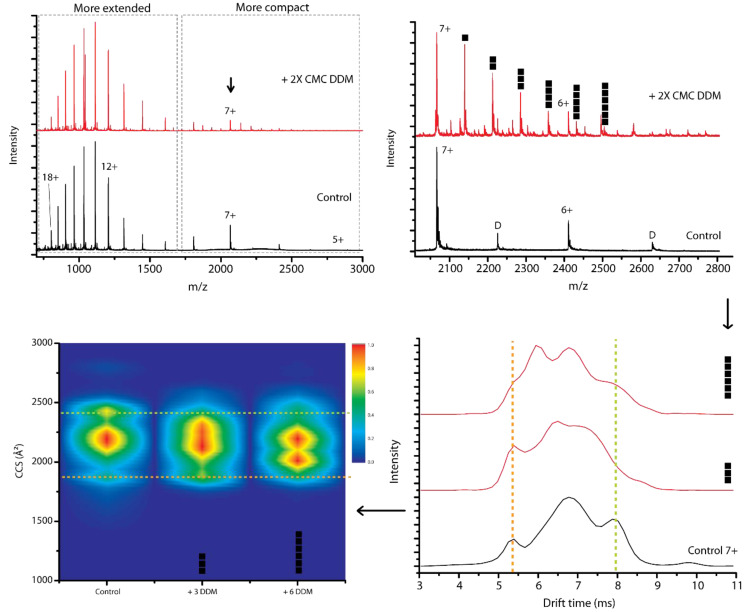
To obtain drift time and CCS values of unbound and detergent bound peaks, we start from the mass spectra shown in the top part of the figure for the control and the mass spectrum with DDM present. Signals that can be attributed to dimers are indicated by “D”. Specific detergent adduct peaks are selected at half height to obtain drift time information. Drift time values (bottom right) are converted to CCS values after calibration and plotted (bottom left) with normalized intensities from 1.0 (highest intensity, red) to 0.0, (not present, dark blue).

Data analysis for mass spectrometry experiments: Calibration is necessary to convert traveling wave drift times to collision cross sections. Ion mobility measurements of α-syn were calibrated using denatured cytochrome C from equine heart, denatured ubiquitin from bovine erythrocytes and denatured myoglobin from equine skeletal muscle (all purchased from Sigma-Aldrich, St. Louis, MO, USA). For each protein a final concentration of 10 µM in 50/49/1 H_2_O/acetonitrile/formic acid was used as travelling wave ion mobility calibrants based on literature CCS values [56]. Acetonitrile (≥99.8%) and formic acid (99%) were purchased from Thermo Fisher Scientific (Waltham, MA, USA). Ion mobility measurements of β-lac were calibrated using native globular proteins: avidin from egg white, bovine serum albumin and cytochrome C from equine heart (all from Sigma Aldrich, St. Louis, MO, USA). For each protein a final concentration of 10 µM in 50 mM ammonium acetate was used as traveling wave ion mobility calibrants, based on literature CCS values [56]. Figure 9 depicts the workflow we applied to extract ion mobility data representing conformational information from mass spectra, illustrated here for the 7+ charge state for the control and DDM adducts. Drift time plots (bottom right panel) can be obtained from individual peaks in the mass spectrum, extracted from the m/z range representing the upper half of each peak. These drift time plots are converted after calibration to CCS plots (bottom left panel), as a way to visualize the conformational space of α-syn monomers. Longer drift times correlate with higher CCS values, shorter drift times with lower CCS values and more compact conformations.

Analysis and visualization of nESI(-IM)-MS data was done using Masslynx V4.1 (Waters Corporation, Wilmslow, UK) and Origin 8.0 (OriginLab Corporation, Northampton, MA, USA). Further analysis of CIU plots was performed using TWIMExtract and CIUSuite 2 [57,58].

## 5. Conclusions

In this study, the first of its kind, we demonstrated that nESI-IM-MS can capture the conformational space of important biological targets which are challenging to study with other, more conventional biophysical techniques. The native mass spectrometry approach used is capable of detecting diverse detergent binding stoichiometries and binding affinities, and investigating the conformational effects which different detergents have on the protein-taking into account the (net) charge and structure of the detergent molecule and its ability to form hydrophobic and/or electrostatic interactions with α-syn.

We found that different types of detergents bind to α-syn, but contrary to expectations their net charge is not the key determinant of binding. Instead the conformational selectivity (low or high α-syn charge states) and binding affinity are determined by the specific molecular structure of the detergent. Since both the free detergent concentration (below CMC) and micelle concentration (above CMC) affect the number of detergents binding to the protein, i.e., the stoichiometry, this indicates that α-syn does interact with free molecules as well as with micelles. Conformational changes were detected when α-syn interacted with different detergent types, and detergent charge was not the primary factor as unique conformational patterns were found for different detergents. Most detergents studied here preferentially bound to more compact conformations, i.e., lower charge states, of α-syn, and compact them further as evidenced by a shift to lower CCS values. Specific conformational states are also stabilized towards unfolding when certain detergents are binding. As α-syn is present in a crowded cellular environment, the observed conformational behavior and cumulative compacting effect of Ca^2+^ as a physiologically important interactor, motivate further work in order to translate our findings to more complex in vivo paradigms.

Using detergents to mimic biological membranes has of course its limitations, but it is an important step to fully understand how interactions between the unstructured protein and more complex systems such as presynaptic membranes occur in vivo. Earlier work on the interaction of α-syn with lipids [59] can now be extended using nESI-IM-MS as a technique capable of concurrently detecting individual conformational states of α-syn present in a heterogeneous ensemble. Future studies will focus on determining α-syn binding regions or sites for detergents, with the aim to engender a more refined classification which informs how certain binding sites for a detergent can result in specific conformational effects. Studying the interaction of α-syn with more complex systems mimicking the biological membrane, e.g., bilayers, nanodiscs or SMALPs with attention to certain oligomeric species that might be formed, will be crucial to further understand the interaction between the protein and biological membranes in vivo.

## Figures and Tables

**Table 1:** Interconversion between × CMC and mM for the standard detergent concentrations and the equal detergent concentrations used. Detergents where concentrations were always above or below their CMC are marked in yellow and grey, respectively. For SDS the concentration increases when going from the standard (0.02× CMC) concentration used to 0.2 mM while for all other detergents here the concentration decreases.

Name	CMC (mM)	Standard Concentration (mM)	Standard Concentration (× CMC)	Equal Concentration (mM)	Equal Concentration (× CMC)
DDM	0.17	0.34	2.0	0.2	1.18
PC-14	0.12	0.24	2.0	0.2	1.67
PC-15 L	0.07	0.14	2.0	0.2	
PC-15 H	0.07	0.24	3.4	0.2	2.86
PC-16 L	0.013	0.026	2.0	0.2	
PC-16 H	0.013	0.24	18.5	0.2	15.38
SDS	8.2	0.164	0.02	0.2	0.024
SCDC	3.0	0.6	0.2	0.2	0.067
SGDC	2.1	0.42	0.2	0.2	0.095

**Table 2:** Overview of the chemical structure of detergents used together with the type, CMC, standard experimental concentration and molecular mass. * The exact molecular mass of Triton X-100 varies as the chain can have a different number of ethylene oxide groups, adding 44 Da for each additional group.

Structure	Name	Type	CMC (mM)	x CMC Used	Molecular Mass (Da)
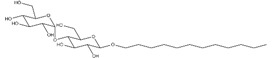	DDM	Non-ionic	0.170	2	510.6
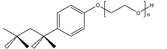	Triton X-100	Non-ionic	0.230	2	650 *
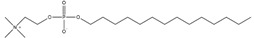	PC-14	Zwitterionic	0.120	2	379.5
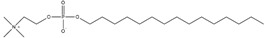	PC-15	Zwitterionic	0.070	2 & 3.4	393.5
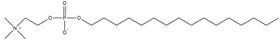	PC-16	Zwitterionic	0.013	2 & 18.5	407.5
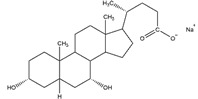	SCDC	Anionic	3.000	0.2	414.6
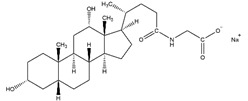	SGDC	Anionic	2.100	0.2	471.6
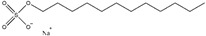	SDS	Anionic	8.200	0.02	288.4
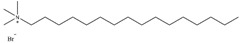	CTAB	Cationic	0.920	0.2	364.5

## Supplementary Materials

Supplementary materials can be found at https://www.mdpi.com/1422-0067/21/21/7884/s1.

## Abbreviations

α-syn	α-synuclein
aa	amino acid
PD	Parkinson’s disease
NAC	Non-Amyloid-β Component
PTM	Post translational modification
SMALP	Styrene maleic acid lipid particle
SUV	Small unilamellar vesicles
CMC	Critical micelle concentration
nESI-IM-MS	Nano-electrospray ionisation ion mobility-mass spectrometry
CCS	Collision cross section
CIU	Collision induced unfolding
β-lac	Β-lactoglobulin
DDM	n-Dodecyl β-D-maltoside
PC-14	n-tetradecylphosphocholine
PC-15	n-pentadecylphosphocholine
PC-16	n-hexadecylphosphocholine
SDS	sodium dodecyl sulfate
SCDC	sodium chenodeoxycholate
SGDC	sodium glycodeoxycholate
CTAB	Cetrimonium bromide
SASA	Solvent accessible surface area
MD	Molecular dynamics
HD	High definition
