# Supplementary material for: Effects of Detergent on α-Synuclein Structure: A Native MS-Ion Mobility Study
Source: Int J Mol Sci. 2020 Oct 23;21(21):7884. doi: 10.3390/ijms21217884 (PMC7660655; doi:10.3390/ijms21217884)
Supplement: Supplementary file 1 [file ijms-21-07884-s001.pdf]

Supplementary information: Effects of detergent on α-synuclein structure. A native MS – ion mobility study

Rani Moons[1,2], Renate van der Wekken-de Bruijne[1], Stuart Maudsley[2], Filip Lemière[1], Anne-Marie Lambeir[3], Frank Sobott[1,4,5,*]

[1] Biomolecular and Analytical Mass Spectrometry group, University of Antwerp, Antwerp, Belgium,
[2] Receptor Biology Laboratory, University of Antwerp, Antwerp, Belgium
[3] Laboratory of Medical Biochemistry, University of Antwerp, Antwerp, Belgium
[4] Astbury Centre for Structural Molecular Biology, University of Leeds, Leeds, United Kingdom
[5] School of Molecular and Cellular Biology, University of Leeds, Leeds, United Kingdom
* Correspondence: F.Sobott@leeds.ac.uk

Interaction of detergents with α-synuclein

As a reference system, detergent binding to the protein β-lac was also investigated and compared to maximum binding stoichiometries that were detected for α-syn. Supplementary Figure 1 shows the maximum number of individual detergent molecules bound to α-syn and β-lac per detergent, with a signal-to-noise ratio S/N ≥ 3. The charge state(s) where this stoichiometry was detected are indicated in the figure per detergent.

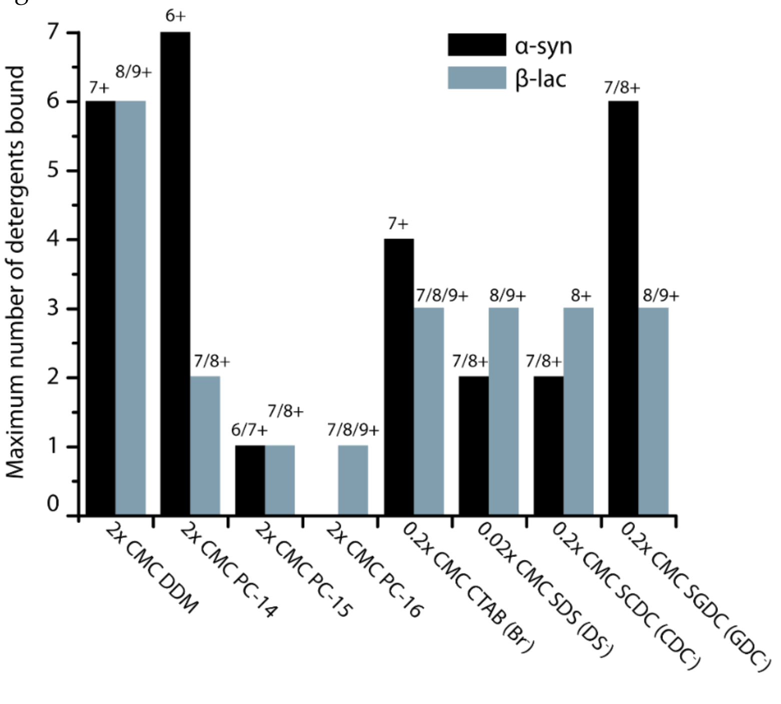

***Supplementary Figure 1:** Maximum binding stoichiometry per detergent for α-syn and β-lac. The charge state(s) where this stoichiometry was detected is indicated.*

Similar to α-syn, β-lac has a high binding capacity for the neutral detergent DDM. β-lac is known to have a hydrophobic cavity where ligands can bind, for example DDM [1]. This might be an indication that the two proteins interact with this detergent in a similar way and mainly through hydrophobic interactions. The binding capacity of β-lac for PC-14 is however significantly lower. In general, for β-lac there seems to be no effect of the chain length of the zwitterionic detergents tested and it binds these detergents poorly. As for α-syn, when CTAB is present only binding of Br^- and not the cationic detergent could be detected to β-lac. For the anionic detergents DS^- and CDC^-, binding capacity seems to be similar for both proteins with up to two molecules bound for α-syn and three for β-lac. β-lac can be embedded

in *e.g.* SDS micelles in a denatured conformation, so it might be that binding of detergent molecules is retained from the micelle rather than individual interacting detergent molecules [2]. For GDC^{-} there is however a significant difference in binding capacity with six molecules bound to α-syn and three for β-lac, the latter is similar to the maximum binding stoichiometry of the other anionic detergents for β-lac.

Detergent binding capacity and stoichiometry

Most of the detergents that were included in this study bind to α-syn and their bound states can be clearly detected. For Triton X-100, however, the intensity of the bound states is very low. Supplementary Figure 2 shows the intensity of bound and unbound states of the 7+ (A) and 12+ (B) charge state of α-syn monomers when 2x CMC Triton X-100 is present (red spectrum). As the mass of Triton X-100 can differ according to the number of ethylene oxide groups in the tail of the molecule, more than one peak can be found for the bound state with one Triton X-100 molecule bound. The difference of 44 Da, one ethylene oxide unit, is indicated by a bow. For the 12+ charge state, the intensity of the bound states is higher compared to the 7+ charge state. For this higher charge state also more peaks that can be attributed to Triton X-100 molecules with different number of ethylene oxide groups are found. This might indicate that the longer the chain of Triton X-100 is, the more it prefers to bind to more extended higher charge states.

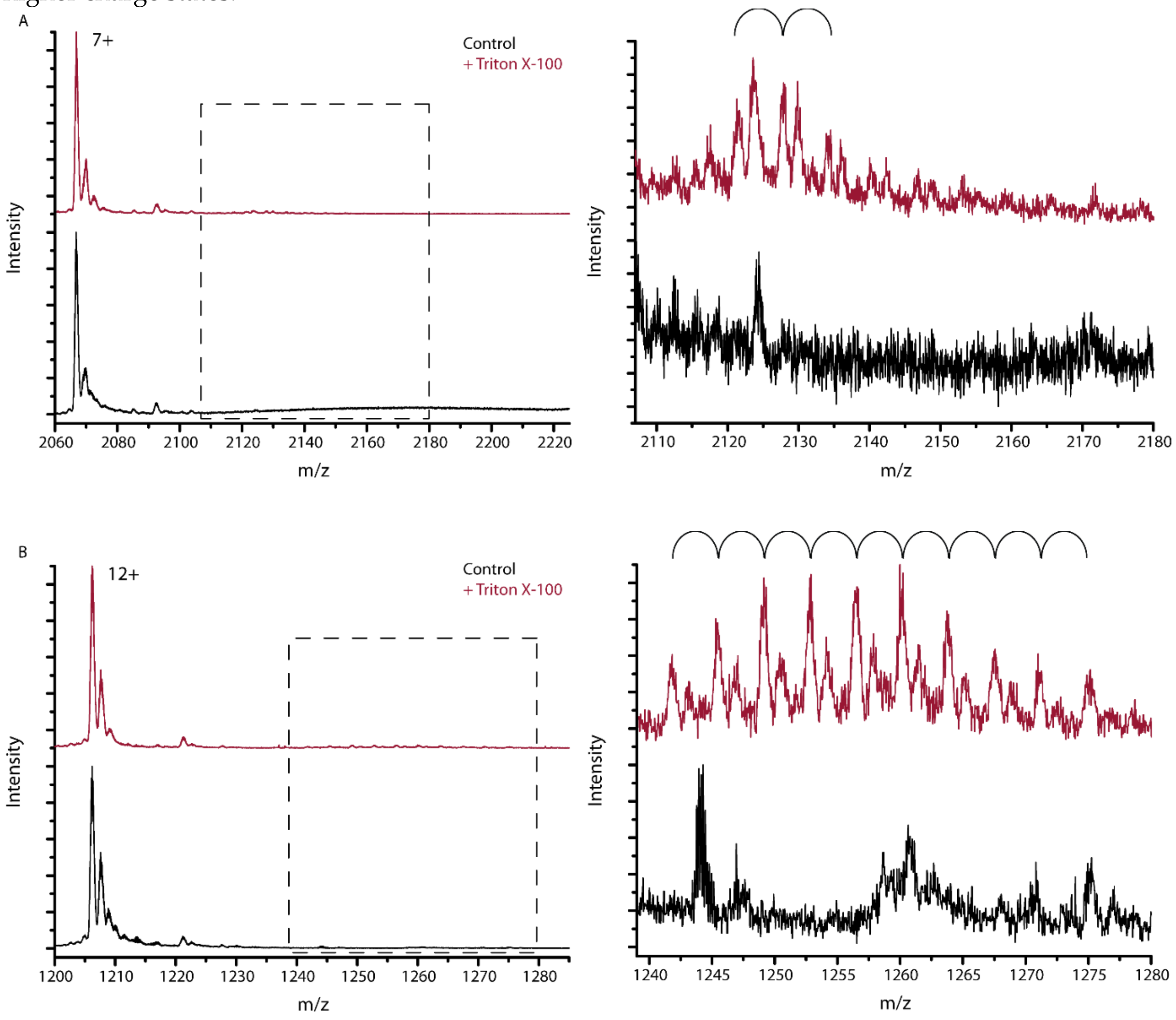

Supplementary Figure 2: *Interaction of Triton X-100 with (A) the 7+ charge state of α-syn and (B) the 12 charge state of α-syn. The α-syn control spectrum is in black, the spectrum in the presence of 2x CMC Triton X-100 in red. At the right, zoomed in spectra of a specific region from the spectra on the left is shown. Mass differences in Triton X-100 corresponding to 44 Da, consistent with one ethylene oxide unit, are indicated by arches.*

When CTAB is added to α-syn or β-lac, no binding can be detected of the intact detergent or the cationic group with detergent properties. Only Br^- is found to bind to both proteins as is shown in Supplementary Figure 3. The top part shows the full MS spectra of α-syn (A) and β-lac (B) without (black line) and with (red line) 0.2x CMC CTAB present. For β-lac the presence of CTAB results in partial denaturation which results in a shift of the charge state distribution, as the charge state range is increased from 6+ to 16+ instead of 6+ to 9+. CTAB is known as a harsh detergent that can disrupt inter- and intramolecular interactions, leading to denaturation [3]. This is similar to what was expected for SDS, however, we did not see similar denaturing effects on β-lac when SDS was present. As α-syn is already partially denatured in its native state, we don't see this denaturing effect of CTAB with α-syn. The bottom part of the figures zooms in on specific parts of the MS spectra and Br^- binding is here indicated with a black square. No peaks apart from dimers (D) and an impurity in the β-lac spectrum (*) can be detected besides Br^- bound peaks. For up to the 11+ charge state of β-lac, Br^- binding can be observed, while for α-syn this is detected for up to the 9+ charge state.

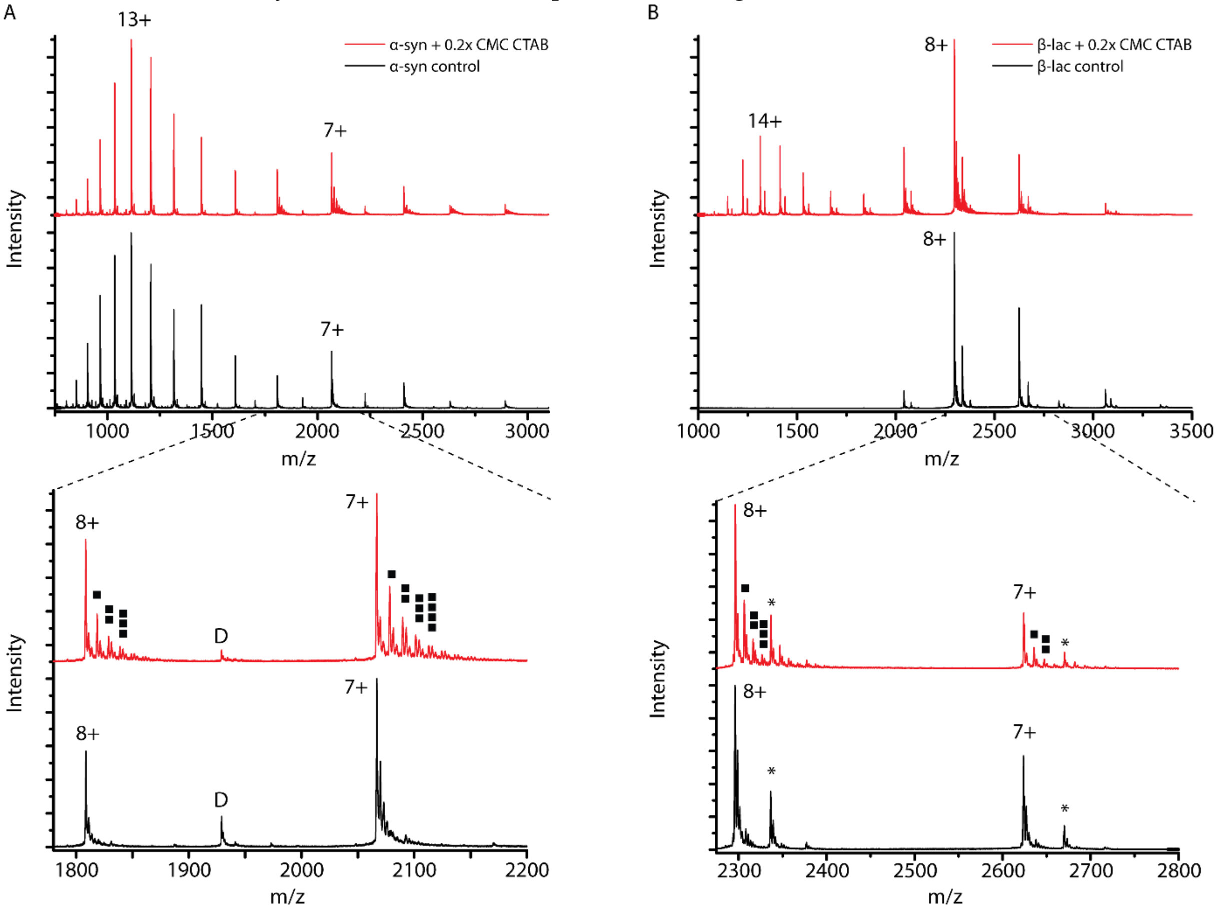

Supplementary Figure 3: *Mass spectra of α-syn (A) and β-lac (B) without (black line) and with (red line) 0.2x CMC CTAB present. The top parts show the full mass spectra, indicating the presence of CTAB induces partial denaturation of β-lac, resulting in the appearance of increased charged states. The bottom part zooms in on specific parts of the mass spectra to show that only* Br^- *binds to both proteins and not CTAB or the cationic part of the detergent. 'D' indicates dimer peaks and '*' is attributed to contaminant peaks in the β-lac spectrum. Black squares indicate* Br^- *binding.*

Conformational selectivity of detergent binding

As can be seen in the previous figure for β-lac, the addition of detergents could lead to a shift in charge state distribution. Supplementary Figure 4 indicates if the intensities of the 7+ and 8+ charge state of α-syn increase or decrease when a specific detergent is present. Every spectrum was first normalised to the intensity of the most intense peak. All stoichiometries (bound and unbound) were then summed and compared to the unbound 7+ or 8+ charge state of the control, respectively, as the 100% reference. In the presence of detergents the range of observed charge states in the mass spectra did not shift, in

each case charge states 5+ or 6+ up to 18+ were detected as was seen in Figure 9 in the main text and in Supplementary Figure 3A. However, the intensities of this distribution are important as well, which is why changes in intensity of the 7+ and 8+ charge state, being seen as the most relevant here as most detergents bind those charge states representing more compact conformations, are further investigated.

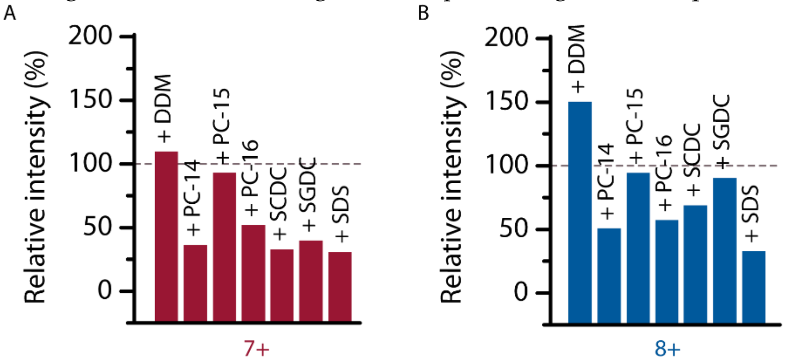

Supplementary Figure 4: *Relative change in intensity (normalised to the intensity of the unbound state of the control (100%) for the respective charge state) for the 7+ (A) and 8+ (B) charge state (unbound + bound states) when initial concentrations of detergents are present.*

DDM is the only detergent that induces a shift in the conformational equilibrium towards the more compact, lower charge states. As DDM binding is charge neutral this is a first indication that detergents are capable of altering the monomer ensemble of α-syn. For zwitterionic detergents the chain length again seems to play an important role as a much stronger decrease in intensity of the lower charge states can be detected for PC-14 and PC-16, compared to a small decrease for PC-15, here all present with a final concentration of 2x CMC. Anionic detergents lower the intensity of the lower charge states as well. Binding of a negatively charged ligand would by itself reduce the charge state and hereby increase the intensity of the lower charge states, but as explained before charge states correlate with the SASA. Protonation can as well compensate ligand charges and these intensity shifts indicate again that the charge of the interacting detergent alone is not sufficient to determine their effects on α-syn monomers and that additional polar and apolar interactions are very important.

Conformational effects of detergent binding

As shown in the main text for the 7+ charge state, here CCS plots are shown of the 6+ charge state (Supplementary Figure 5) and the 8+ charge state (Supplementary Figure 6) to visualize conformational effects when detergent molecules bind to α-syn monomers. Binding of DDM is observed for the 8+ charge state and, as was the case for the 7+ charge state, leads to an intensity shift towards more compact charge states. For the zwitterionic detergent, PC-14 binding is also observed for both charge states, however, with a very high number bound to the 6+ charge state as was discussed in Figure 2 in the main text. PC-15 and PC-16 binding is detected for the 6+ charge state but not for the 8+ charge state. The exact conformational trend for these zwitterionic detergents, compaction or elongation, is a bit dubious as for the 6+ states very low intense more compact conformations seem to occur for PC-15 and PC-16, but not for PC-14. In the latter case it seems there is almost no conformational effect occurring apart from increasing CCS values likely due to the additional surface area of the detergent molecules themselves. The three negatively charged detergents all show a very clear compacting trend for the 8+ charge state, DS$^-$ and CDC$^-$ have a very clear conformational effect just being present in the sample indicating a memory effect of previously bound ions that were lost during the measurement as seen for the 7+ charge state. GDC$^-$ only has an effect when one or more molecules are bound to the protein so here we don't see this memory effect. For the 6+ charge state the only anionic detergent of which binding was detected is DS$^-$, which didn't lead to a conformational effect. In general, it is likely that the 6+ charge state is already a very compact and possibly partly gas phase collapsed form, resulting in very little to none conformational effects when detergent molecules interact with these protein conformations.

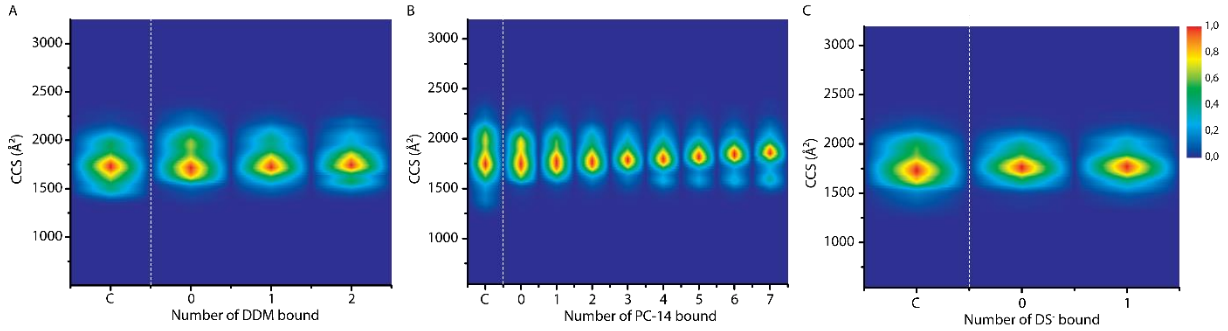

Supplementary Figure 5: *CCS plots of the 6+ charge state for (A) DDM, (B) PC-14 and (C) DS- stoichiometries. This charge state is not very sensitive to conformational detergent effects likely because of its already very compact conformation.*

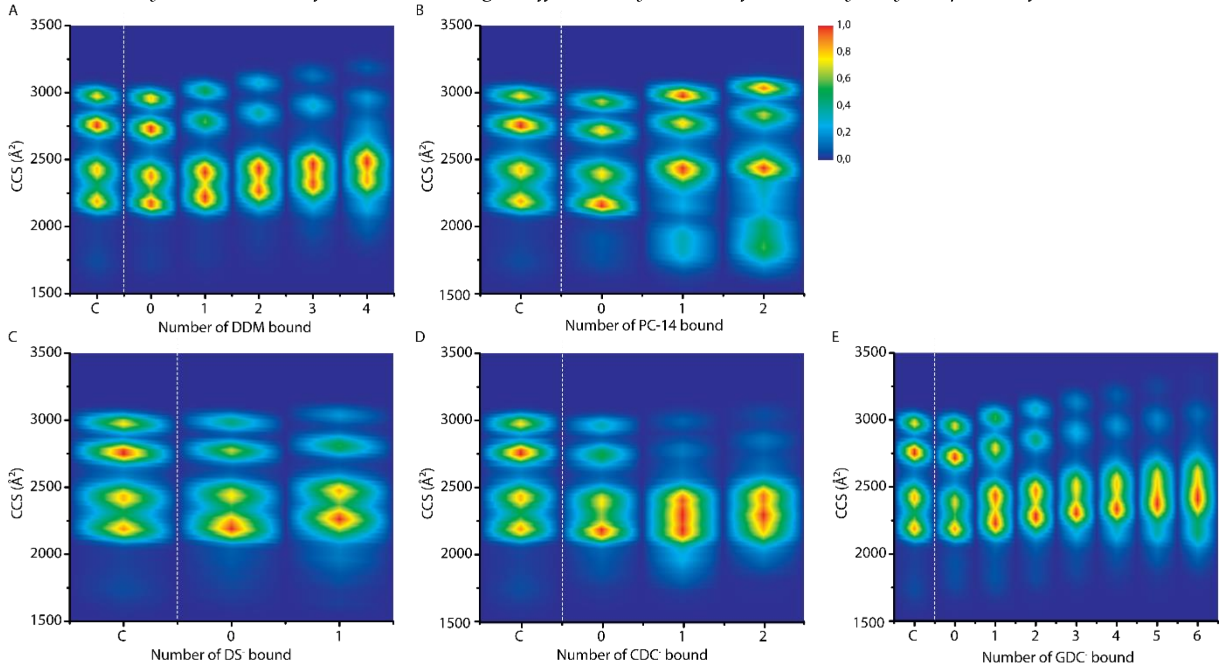

Supplementary Figure 6: *CCS plots of the 8+ charge state for different detergent stoichiometries per detergent. Here very clear conformational effects can be detected resulting in intensity shifts and a unique CCS pattern.*

After CCS plots representing the lower charge states and hereby the more compact protein conformations, Supplementary Figure 7 shows the CCS plot of the 11+ charge state of α-syn when DDM binds. "C" again represents the unbound 11+ charge state without any detergent present, "0" represents the unbound state but with DDM present in the sample and higher numbers indicate the number of DDM molecules bound. As was seen for the lower charge states, a slight increase in CCS values can be detected when more DDM molecules bind, related to the additional volume of those detergent molecules. No other conformational effects can be detected. For higher charge states, Coulombic forces between the charges increase resulting in more extended conformations. Because of these forces it becomes more difficult for structural interactors, such as detergent molecules, to alter the conformational ensemble of the protein. This is why we are more focussed on the lower charge state region.

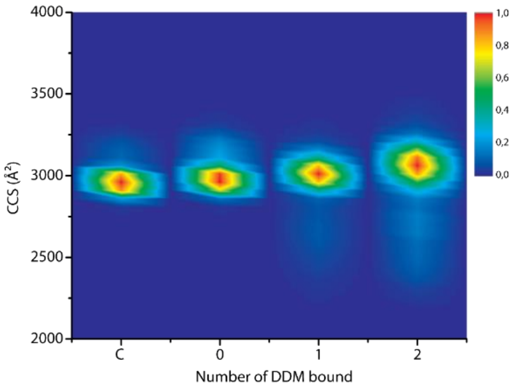

Supplementary Figure 7: *CCS plot of the 11+ charge state of α-syn without detergent present "C", with detergent present but not bound "0" and with a specific number of DDM bound indicated by the number.*

Supplementary Figure 8 shows CCS plots of the 7+ charge state of β-lac with one of three types of detergents present (DDM, PC-14 and DS⁻) and binding to the protein to see if detergent binding also results in conformational changes in the case of β-lac. While clear conformational shifts could be observed for α-syn, no shift in CCS values is detected for β-lac when DDM, PC-14 or DS⁻ are bound to the protein. This indicates that the conformational effects we see for α-syn are specific for the interaction between detergents and that IDPs are more sensitive, compared to globular proteins, to conformational changes as a result of interactions with detergents.

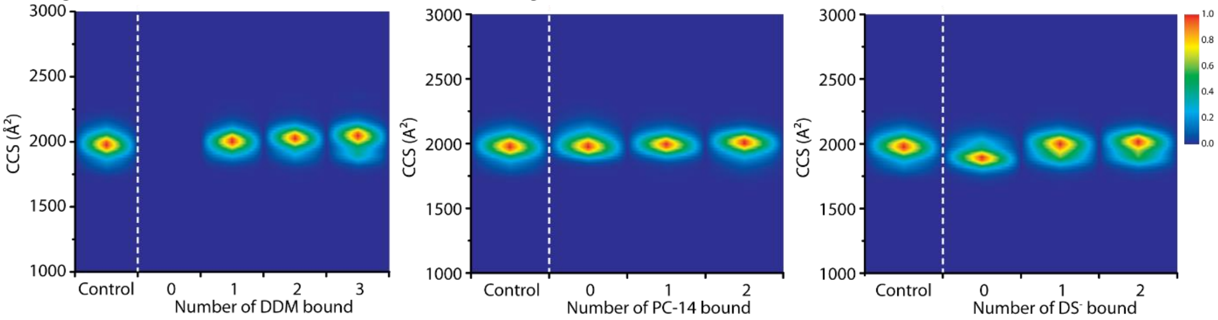

Supplementary Figure 8: *Conformational effect of DDM, PC-14 and SDS on the 7+ charge state of β-lac. No conformational change can be detected.*

As it is known that Ca^{2+} plays an important role in the interaction between biological membranes and α-syn, the conformational effect on the protein is investigated when both a detergent and Ca^{2+} are present. CCS plots of the 7+ charge state of α-syn are shown in Supplementary Figure 9. In panel A only $CaCl_2$ is present in a 1:20 protein to metal ion ratio as a control, in panel B $CaCl_2$ and 2x CMC PC-14 are present, in panel C $CaCl_2$ and 0.2x CMC SCDC are present and able to interact with α-syn.

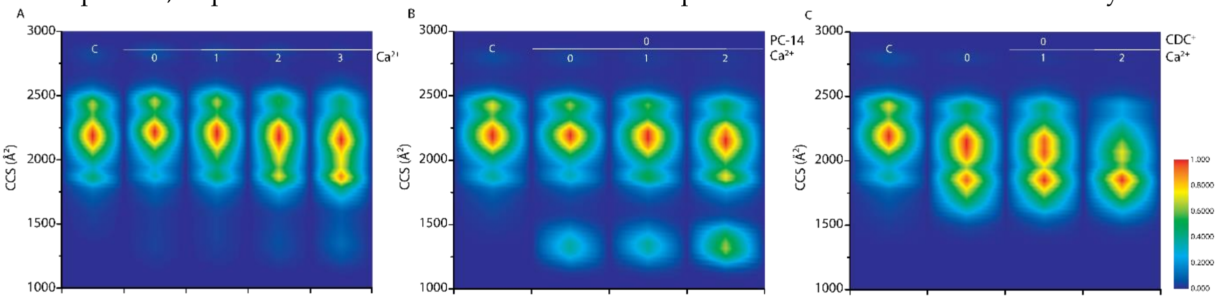

Supplementary Figure 9: *Conformational effect of Ca^{2+} in addition to effects of the detergent present in the sample, binding to the 7+ charge state of α-syn. (A) Ca^{2+}, (B) 2x CMC PC-14 and Ca^{2+} and (C) 0.2x CMC SCDC and Ca^{2+}.*

In the control panel A a gradual compaction when more Ca^{2+} ions bind, with a maximum of three ions bound, is observed as described in earlier studies [4,5]. It can be seen that for PC-14, the binding of additional Ca^{2+} ions together with detergent molecules leads to the formation of additional very compact conformations as was seen for DDM in Figure 3F in the main text. However, the resulting conformational pattern is different which indicates a specific effect related to a certain detergent. When CDC^- binds, there is an intensity shift towards more compact conformations however only conformations which were already present before. This indicates that for neutral and zwitterionic detergents there seems to be a cumulative compacting effect of the detergent and Ca^{2+}. This might be linked to different binding sites on the protein for the detergent and Ca^{2+} ions, both leading to local compacting effects. For the anionic detergent an intensity shift is again already detected for the unbound "0" state, indicating that detergent and/or Ca^{2+} ions might be lost along the way. The number of detergents that can bind to the protein is not affected by the presence of Ca^{2+} and vice versa.

Probing conformational transitions

In Supplementary Figure 10 is the drift time plot shown where the major conformational families are indicated that are present for the 7+ charge state of α-syn without detergent present. Additionally the CIU50 value, the midpoint of the transition between the most intense two features (1a and 2a), was calculated at 22.1 V. When performing a Gaussian fitting of the drift time profile of the data point before and after the transition (trap CE 20 V and 25 V, respectively), four different conformational families are detected. Besides the two major features already discussed for panel A (1a and 2a) there is an additional very compact state (1b) and an additional more extended state (2b), of which intensities depend on the trap CE voltage and also indicated in all panels.

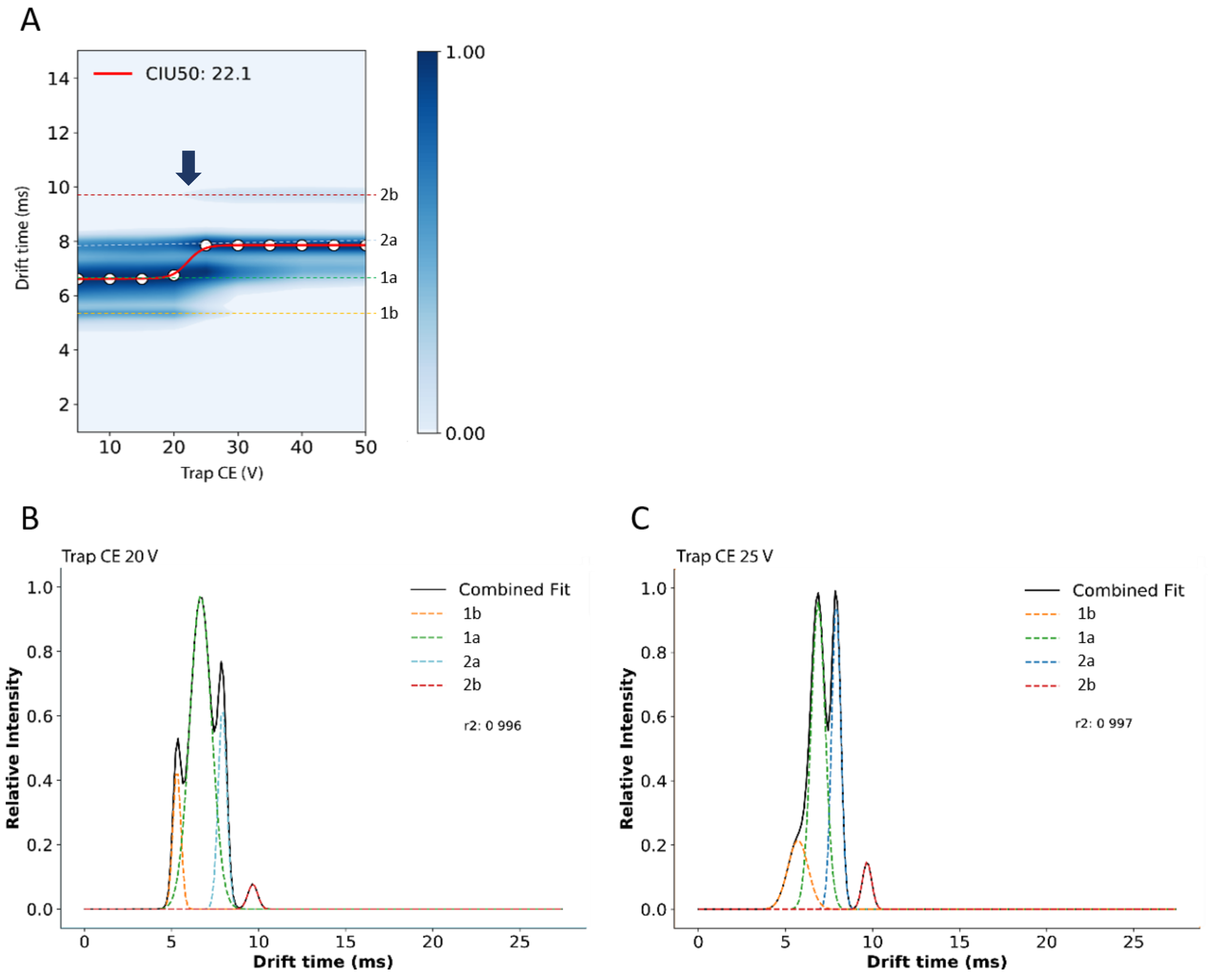

Supplementary Figure 10: *Analysis of the 7+ charge state CIU plot of α-syn without detergent present. (A) Two major features in the conformational ensemble are detected (1a and 2a) and hereof the CIU50 value, the midpoint of the transition*

between two features, was calculated using CIUsuite 2 at 22.1 V. (B) and (C) show Gaussian fits of the drift time plots with a Trap CE of 20 V and 25 V respectively, as these are the datapoint before and after conformational transition. Besides the two major features (1a and 2a), two additional conformations are found (1b and 2b) as can also be seen on the plot in A and the CIU plot in the main text in Figure 4.

As was done for the 7+ charge state in the main text, Supplementary Figure 11 shows CIU plots of the 8+ charge state of α-syn without (control) and with detergents that affect stability of certain conformations. The control plot shows four distinct conformational families, as found in earlier studies [4,6,7]. The x-axis of each plot indicates up to which voltage binding of one detergent molecule was detected with S/N ≥ 3.

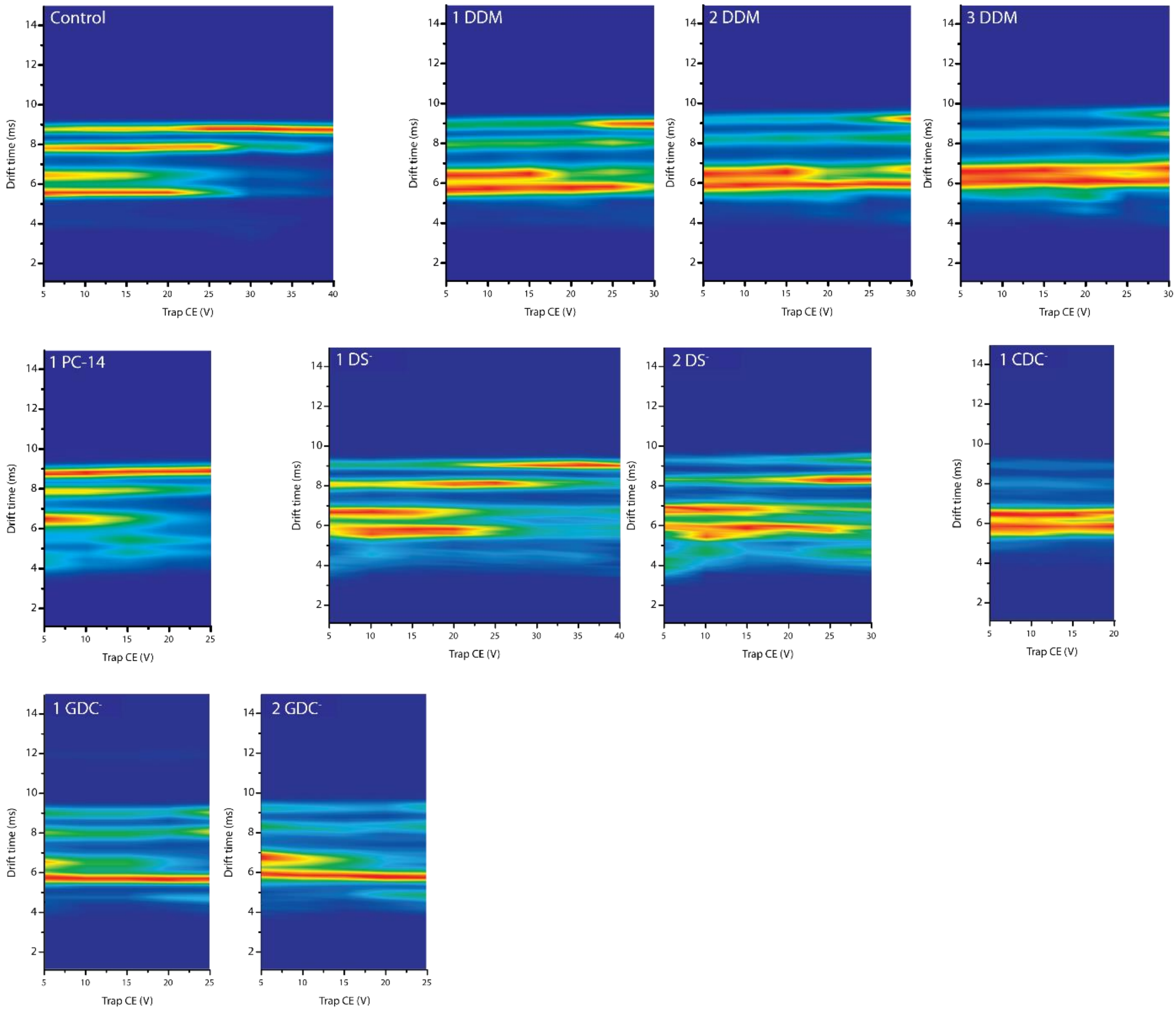

Supplementary Figure 11: *CIU plots of the 8+ charge state of α-syn when a specific detergent is bound with a specific stoichiometry.*

For DDM a stabilisation of the two most compact conformational families can be observed with the most compact conformation being present for voltages up to 25 V and the second most compact conformation having a high intensity until 15 V while this isn't the case in the control. Stability of these compact conformations is increased when more DDM molecules bind to the protein, with three DDM molecules present both compact conformations are still present when detergent binding is lost at 30 V. This trend is similar to what we saw for the 7+ charge state. For PC-14, of which the protein-detergent interaction was broken at too low trap CE voltages to be able to detect any stabilising effects for the 7+ charge state, we see a destabilisation of the three most compact conformational families for the 8+ charge state. As it wasn't clear from the CCS plots alone if and which conformational effects could occur when this

detergent is present, it is interesting to see that it has a preference for stabilising of the more extended conformation. In contrast to the drastic stabilizing effect that was seen for the 7+ state, DS$^-$ does not seem to significantly stabilize more compact conformations of the 8+ charge state. Both other anionic detergents, CDC$^-$ and GDC$^-$ do seem to result in stabilisation of the more compact conformational families, especially the most compact one in the case of GDC$^-$, although detergent binding is lost at relative low trap CE voltages, making it difficult to completely confirm this effect.

Detergent concentration effects

In Supplementary Figure 12 CCS plots are compared of DDM, PC-14, CDC$^-$ and GDC$^-$ binding to α-syn when the final detergent concentration is either the standard experimental concentration used (see Tables 1 and 2, main text) or is equal to 0.2 mM. There doesn't seem to be a significant conformational effect related to the final detergent concentration in solution for these detergents. For PC-14 and GDC$^-$ binding no difference in CCS values are detected for a certain stoichiometry when different detergent concentrations are present. For DDM there is a slight delay in the compaction observed with three and four detergent molecules bound when 0.2 mM DDM is present. For CDC$^-$ binding however, there seems to be a slight delay on the compacting effect when 0.2x CMC is present. Conformational effects are for all detergents mainly related to the binding stoichiometry and not to the free individual detergent or micelle concentration in the sample.

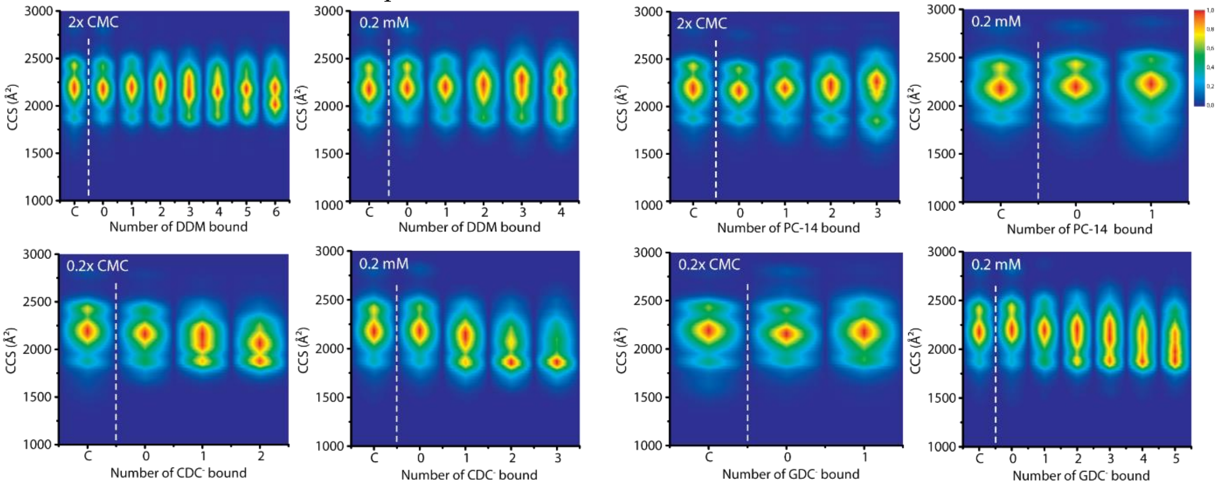

Supplementary Figure 12: *Comparison of CCS plots when the standard concentration of detergent was present and when 0.2 mM of detergent was present.*

In Supplementary Figure 13 CCS plots are shown of the different binding stoichiometries for the 7+ state of α-syn when SDS is present in the sample with a final concentration of 0.02x CMC (left panel) and 0.2 mM (right). In both cases there is an intensity shift towards more compact conformations when DS$^-$ binds as discussed earlier. This conformational shift occurs with less DS$^-$ ions bound when a lower concentration of SDS is present in comparison to when a higher concentration of SDS is present. This would indicate that one specific DS$^-$ binding stoichiometry does not result in one specific conformational pattern. To explain this, we hypothesize that memory effects can play an important role here. If originally more detergents were binding to the protein when a final concentration of 0.02x CMC is present, but are not retained during the measurement, we can still pick up conformations which originally came from complexes with higher binding stoichiometries. The fact that for the "0" bound

state there is already a conformational effect on the left panel, while this shouldn't be the case as nothing is interacting with the protein, supports this hypothesis.

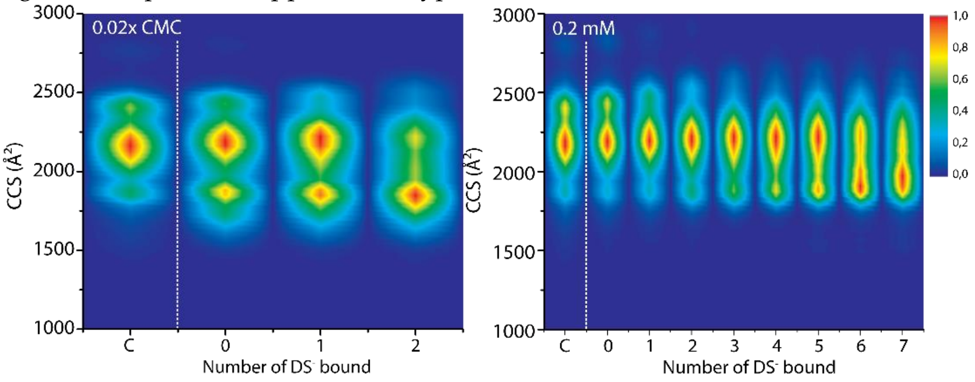

Supplementary Figure 13: *Comparison of CCS plots of the 7+ charge state of α-syn when the initial concentration of detergent was present (left) and when 0.2 mM of detergent was present (right).*

Effect of chain length within the same detergent class

In Supplementary Figure 14 an eventual shift in intensity of the lower charge states of α-syn is investigated when zwitterionic detergents are present and bound. The intensity of the unbound charge state of α-syn without any detergent present serves as the reference with an intensity of 100%. Results are shown of samples containing a final concentration of 2x CMC PC-14, PC-15 (PC-15 L) or PC-16 (PC-16 L) and samples with a final detergent concentration all equal to 2x CMC PC-14, which is 3.4x CMC PC-15 (PC-15 H) or 18.5x CMC PC-16 (PC-16 H). For the 7+ and 8+ charge states, all zwitterionic detergents tested here result in a decrease of intensity for these charge states. These shifts might be due to conformational changes of the protein when these zwitterionic detergents are present, resulting in intensity shifts in the charge state distribution. In order to establish whether this happens, IM-MS experiments are performed to detect and study eventual conformational changes in more detail (see Figure 8 in the main text).

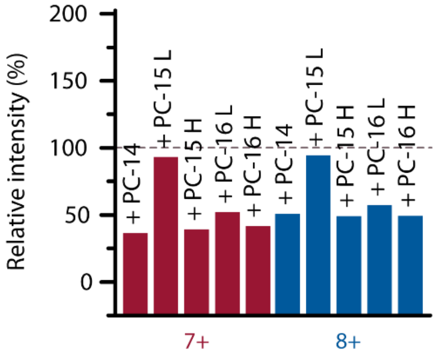

Supplementary Figure 14: *Relative differences in intensity of the lower charge states of α-syn when detergent is present compared to the control without detergent present (unbound charge state control = 100%). "L" indicates 2x CMC, "H" indicates 0.24 mM or 2x CMC PC-14.*

References

1 Hansted, J. G., Wejse, P. L., Bertelsen, H. & Otzen, D. E. Effect of protein-surfactant interactions on aggregation of beta-lactoglobulin. *Biochim Biophys Acta* **1814**, 713-723, doi:10.1016/j.bbapap.2011.03.011 (2011).

2 Dominguez-Ramirez, L., Del Moral-Ramirez, E., Cortes-Hernandez, P., Garcia-Garibay, M. & Jimenez-Guzman, J. beta-lactoglobulin's conformational requirements for ligand binding at the calyx and the dimer interphase: a flexible docking study. *PLoS One* **8**, e79530, doi:10.1371/journal.pone.0079530 (2013).

3 Seddon, A. M., Curnow, P. & Booth, P. J. Membrane proteins, lipids and detergents: not just a soap opera. *Biochim Biophys Acta* **1666**, 105-117, doi:10.1016/j.bbamem.2004.04.011 (2004).

4 Stephens, A. D. *et al.* Extent of N-terminus exposure of monomeric alpha-synuclein determines its aggregation propensity. *Nat Commun* **11**, 2820, doi:10.1038/s41467-020-16564-3 (2020).

5 Moons, R. *et al.* Metal ions shape α-synuclein. *Scientific Reports*, doi:10.1038/s41598-020-73207-9 (2020).

6 Daniels, M. J. *et al.* Cyclized NDGA modifies dynamic alpha-synuclein monomers preventing aggregation and toxicity. *Sci Rep* **9**, 2937, doi:10.1038/s41598-019-39480-z (2019).

7 Ponzini, E. *et al.* Methionine oxidation in alpha-synuclein inhibits its propensity for ordered secondary structure. *J Biol Chem* **294**, 5657-5665, doi:10.1074/jbc.RA118.001907 (2019).
